# ${\dot{V}}_{O_{2\max}}$‐based all‐out aerobic exercise attenuates hexosamine biosynthetic pathway activity: Metabolomic insights in mice model

**DOI:** 10.1113/EP093528

**Published:** 2026-06-11

**Authors:** Gyumin Kang, Eui‐Cheol Shin, Jae Kyeom Kim, Young Jun Kim

**Affiliations:** ^1^ Department of Food Regulatory Science Korea University Sejong Republic of Korea; ^2^ Institute of Natural Sciences Korea University Sejong Republic of Korea; ^3^ Department of GreenBio Science (BK21) Gyeongsang National University Jinju Republic of Korea; ^4^ Department of Food and Biotechnology Korea University Sejong Republic of Korea; ^5^ Department of Health Behavior and Nutritional Science University of Delaware Newark, DE USA; ^6^ BK21 FOUR Research Education Team for Omics‐Based Bio‐Health in Food Industry Korea University Sejong Republic of Korea

**Keywords:** all‐out aerobic exercise, glucose metabolism, hexosamine biosynthetic pathway, insulin sensitivity

## Abstract

Aerobic exercise enhances physiological performance by activating metabolic systems, including glucose oxidation and fat metabolism. Cellular glucose predominantly flows through glycolysis (95–98%), whereas a smaller fraction (2–5%) enters the hexosamine biosynthetic pathway (HBP), generating UDP‐*N*‐acetylglucosamine. Excessive carbohydrate intake and inactivity expedite glucose redirection toward the HBP via mass‐action mechanisms, which impair glucose uptake and insulin signalling. Although exercise intensity is a key determinant of fuel selection, with lipid oxidation predominating at moderate intensities (25–40% ${\dot{V}}_{O_{2\max}}$) and glucose utilization becoming the primary energy source during vigorous activity (75–85% ${\dot{V}}_{O_{2\max}}$), the mechanisms by which exercise intensity regulates HBP activity remain poorly understood. To investigate this, 12 male C57BL/6 mice were randomly assigned to control (CG, *n* = 6) and aerobic exercise groups (AEG, *n* = 6), with AEG undergoing 8 weeks of ${\dot{V}}_{O_{2\max}}$‐based all‐out aerobic exercise (∼65.72% ${\dot{V}}_{O_{2\max}}$). Untargeted plasma metabolomics was performed using ultra‐high performance liquid chromatography–time‐of‐flight mass spectrometry and processed using apLCMS, xMSanalyzer, xMSannotator, MetaboAnalyst 6.0 and the KEGG database. A two‐component partial least squares discriminant analysis model achieved 100% classification accuracy with strong explanatory and predictive performance (*R* ^2^ = 0.981, *Q* ^2^ = 0.673). Eight *N*‐acetyl compounds were significantly decreased in AEG compared with CG (*P* = 0.0139; log_2_ fold change = −1.17), corresponding to an approximate 0.445‐fold decrease in AEG relative to CG, with amino sugar and nucleotide sugar metabolism being the sole significantly affected pathway (*P* = 0.0489). Four HBP intermediates (*N*‐acetyl‐d‐glucosamine 6‐phosphate, *N*‐acetyl‐α‐d‐glucosamine 1‐phosphate, *N*‐acetyl‐d‐mannosamine 6‐phosphate, *N*‐acetyl‐α‐d‐galactosamine 1‐phosphate) showed discriminatory performance (AUC = 0.833). All‐out aerobic exercise suppresses HBP activity by lowering its key metabolic intermediates, suggesting a clear shift in glucose flux toward glycolysis. This metabolic redirection likely serves as a protective mechanism against the development of insulin resistance and diabetes.

## Abstract (highlights)

- **What is the central question of this study?**
  This study investigated the metabolic effects of chronic ${\dot{V}}_{O_{2\max}}$‐based all‐out aerobic exercise on glucose‐associated pathways, particularly glycolysis and the hexosamine biosynthetic pathway (HBP), and examined how this intervention reshapes plasma biomarkers related to carbohydrate metabolism.
- **What is the main finding and its importance?**
  Eight‐week ${\dot{V}}_{O_{2\max}}$‐based aerobic exercise markedly suppressed activity in the HBP in mice. Eight *N*‐acetyl metabolites and four key HBP intermediates were significantly reduced, showing strong discriminatory power (AUC = 0.833). Given that elevated HBP flux is mechanistically implicated in the pathogenesis of insulin resistance and type 2 diabetes, the observed downregulation of HBP‐related metabolites suggests that aerobic exercise may redirect glucose metabolism away from the HBP toward glycolytic pathways, thereby providing molecular evidence for exercise‐induced metabolic reprogramming.

## 1 INTRODUCTION

Physical activity is defined as planned, structured and repetitive bodily movements designed to enhance physical fitness and performance (Caspersen et al., 1985). Exercise science categorizes physical activities into two primary metabolic types: aerobic and anaerobic exercise (Chamari & Padulo, 2015). Anaerobic exercise primarily utilizes the glycolytic pathway to generate ATP (Baker et al., 2010), while aerobic exercise engages multiple metabolic processes, including glycolysis, the TCA cycle, electron transport chain (ETC) and lipid oxidation (Baker et al., 2010). Aerobic exercise is characterized by alternating movement patterns and continuous bodily motion that create varying exercise intensities from light to heavy, where maximum oxygen uptake (${\dot{V}}_{O_{2\max}}$) serves as the physiological metric for quantifying these intensity levels. At specific exercise intensities, metabolic pathways that oxidize fats and carbohydrates are engaged concurrently, with their relative contributions shifting reciprocally depending on exercise intensity (Spriet, 2014); fat oxidation predominates at lower intensities (25–40% of maximal ${\dot{V}}_{O_{2\max}}$), while carbohydrate metabolism utilizing plasma glucose and muscle glycogen becomes significantly active during high‐intensity aerobic exercise (75–85% of maximal ${\dot{V}}_{O_{2\max}}$) (Romijn et al., 1993; van Loon et al., 2001). Therefore, even within a single aerobic exercise modality, the intensity level itself represents a critical physiological determinant that can modulate various metabolic pathways and biomarkers. Given that glycolysis remains essential during aerobic activities, particularly during the onset of physical activity (Baker et al., 2010; Hargreaves & Spriet, 2020), it is therefore important to examine the specific alterations in glucose‐associated biomarkers and metabolic pathways induced by chronic aerobic training. In this sense, particular attention should be directed toward competing pathways such as the hexosamine biosynthetic pathway (HBP), whose exercise‐induced modulation may offer mechanistic insights into glucose metabolism and metabolic health. Previous research has predominantly focused on lipid‐related parameters such as total cholesterol (Thorogood et al., 2011), triglycerides (García‐Hermoso et al., 2018; Thorogood et al., 2011; Wewege et al., 2018) and high‐density lipoprotein (García‐Hermoso et al., 2018; Wewege et al., 2018), given that improved lipid oxidation is considered the principal physiological benefit of aerobic exercise (Baker et al., 2010). This emphasis on lipid metabolism has resulted in relatively limited investigation of glucose‐related metabolic adaptations, and the differences between exercise‐induced changes and baseline conditions in untrained or sedentary individuals therefore remain a significant research gap.

In vivo studies have demonstrated that high‐sugar diets using monosaccharides (glucose and fructose) (Musselman et al., 2019) and disaccharides (sucrose) (Palanker et al., 2011) are associated with obesity and diabetes (Musselman et al., 2019; Palanker et al., 2011). Most cellular glucose undergoes glycolysis (Ma & Hart, 2013; Schleicher & Weigert, 2000), whereas 2–5% enters the HBP (Hawkins et al., 1997; Marshall et al., 1991; Traxinger & Marshall, 1991), an alternative branch pathway of glycolysis that produces UDP‐*N*‐acetyl‐α‐d‐glucosamine (UDP‐GlcNAc) (Hart et al., 2007; Torres & Hart, 1984). Excessive glucose intake exceeding the metabolic capacity of glycolysis elevates glucose flux to the HBP through mass action, eventually causing compensatory reduction of glucose transport to biological tissues via insulin action (Traxinger & Marshall, 1991). In turn, increased glucose flux toward the HBP leads to impaired glucose transport (Garvey et al., 1987; Traxinger & Marshall, 1991) and hyperglycaemia‐driven activation of the HBP contributes to insulin resistance (Crook et al., 1993; Giaccari et al., 1995; Robinson et al., 1993; Traxinger & Marshall, 1991). Studies have examined the implications of different sugar types in metabolic syndromes (Musselman et al., 2019; Palanker et al., 2011), but exercise interventions were not feasible in these studies due to the use of *Drosophila* as the experimental model. Likewise, other mouse studies investigated the effects of monosaccharides on glucose and lipid metabolism (Bouwman et al., 2020; Yuan et al., 2020) but did not incorporate exercise interventions. However, it is important to note that aerobic exercise intervention improves insulin sensitivity (Bajpeyi et al., 2009; Donges et al., 2013; Gregory et al., 2019; Slentz et al., 2009) and alleviates insulin resistance (Garcia‐Hermoso et al., 2014; Slentz et al., 2011). Considering the glucose occupancy rates in glycolysis (approximately 95–98%) and the HBP (approximately 2–5%) (Hawkins et al., 1997; Marshall et al., 1991; Traxinger & Marshall, 1991), aerobic exercise may stimulate glycolytic activity, enhancing glucose utilization and oxidation while potentially reducing glucose flux to the HBP. Therefore, investigating how aerobic exercise modulates glucose partitioning between glycolysis and the HBP may provide critical mechanistic insights into exercise‐induced improvements in glucose homeostasis and metabolic health.

Although previous studies have explored metabolic syndromes (Musselman et al., 2019; Palanker et al., 2011) and glucose metabolism (Bouwman et al., 2020; Yuan et al., 2020), experimental limitations often precluded the implementation of standardized exercise protocols or precise intensity controls. Consequently, the specific impact of intensity‐defined aerobic exercise on glucose partitioning between glycolysis and branching pathways such as the HBP has yet to be fully characterized. To this end, we applied untargeted metabolomics using ultra‐high‐performance liquid chromatography (UHPLC) equipped with time‐of‐flight (TOF) mass spectrometry. The primary purpose of the current study is to elucidate plasma metabolic changes following an 8‐week ${\dot{V}}_{O_{2\max}}$‐based all‐out aerobic exercise (VAAE) programme, which focuses on metabolic up‐ and downregulation of relevant biomarkers in carbohydrate metabolism. To ensure robust biomarker discovery, we employed an integrated bioinformatics workflow to identify and validate metabolic signatures associated with the 8‐week exercise programme. This pipeline utilized several R packages, including apLCMS, xMSanalyzer, and xMSannotator, in conjunction with MetaboAnalyst 6.0 and the KEGG Pathway Database (Kanehisa & Goto, 2000).

## 2 METHODS

### 2.1 Ethical approval

All experimental procedures involving animals were approved by the Institutional Animal Care and Use Committee (IACUC) of Korea University (approval number: KUIACUC‐2021‐0017) and conducted in accordance with the Korea Animal Protection Act and institutional guidelines for animal welfare. This work conforms to the ethical requirements outlined by the journal. Animals were housed under controlled environmental conditions (12‐h light–dark cycle, temperature 22 ± 2°C, relative humidity 50–60%) with ad libitum access to standard laboratory chow and water.

### 2.2 Animal subjects and experimental conditions

Twelve male C57BL/6 mice (5‐week‐old) were procured from Samtako (Osan, Republic of Korea) and randomly allocated into two experimental groups: a control group (CG, *n* = 6) and an aerobic exercise group (AEG, *n* = 6). Subsequently, animals were housed with three mice per cage based on randomized assignment protocols. Environmental housing conditions were maintained under a standardized 12:12‐h light:dark cycle (light phase: 07.00–19.00 h; dark phase: 19.00–07.00 h) within a controlled windowless breeding facility maintained at 23–25°C. All subjects were provided with ad libitum access to water and standard laboratory chow, which were replenished as needed to ensure continuous availability. The nutritional composition of the laboratory diet (Envigo 2018S Diets, Envigo, Livermore, CA, USA) comprised 44.2% carbohydrates and 6.2% fat among total micronutrients, ensuring that the dietary formulation was experimentally appropriate for investigating carbohydrate‐related metabolic processes.

### 2.3 Exercise intervention programme and implementation

During the initial 1‐week environmental adaptation period, both groups received only standard nutritional provisions (chow and water) without exercise intervention. Subsequently, throughout the 2‐week exercise adaptation phase, CG maintained identical experimental conditions as established during the environmental adaptation period. AEG underwent a structured 2‐week exercise adaptation protocol with progressively demanding training characteristics. During the first week of adaptation, animals commenced treadmill running without electrical stimulation, beginning at 10.0 m/min (10° inclination) for the first 5 min of each 15‐min session. Treadmill velocity was then progressively increased by 1.0 m/min increments over the subsequent 10 min until it reached a maximum speed of 20.0 m/min. In the second week of adaptation, AEG followed the identical speed progression protocol performed during the first week, with the critical addition of a 1‐mA electric shock applied to the grid to ensure consistent running performance among animal subjects. Throughout the entire 2‐week adaptation period, intervention sessions were conducted three times weekly. Following the exercise adaptation phase, a 6‐week primary exercise intervention was implemented. AEG mice performed identical aerobic exercise protocols as established during the second week of adaptation, maintaining the same three‐times‐weekly frequency. During this 6‐week intervention period, CG animals were housed with access to standard nutritional provisions (chow and water) without an exercise programme throughout the equivalent time frame.

### 2.4 Estimation of physiological exercise intensity in animal model

Since direct ${\dot{V}}_{O_{2\max}}$ measurement was not conducted in our study, exercise intensity was estimated based on established evidence from methodologically comparable research. Previous research demonstrated that treadmill exercise at 20 m/min with 10° inclination in 4‐week‐old untrained male Wistar rats corresponded to 65.72% of maximal ${\dot{V}}_{O_{2\max}}$ (approximately 69.1 mL/kg/min) (Qin et al., 2020). Notably, this study employed nearly identical exercise conditions to our experimental protocol, and the age difference between their 4‐week‐old subjects and our 5‐week‐old mice was minimal, which provides a highly relevant reference for exercise intensity quantification. The establishment of quantified exercise intensity standards is crucial for ensuring reproducibility and comparability across exercise intervention studies. Given that AEG mice struggled to sustain continuous running performance at these parameters, the estimated exercise intensity inducing ‘all‐out’ physiological state likely represents 65.72% of ${\dot{V}}_{O_{2\max}}$ based on this methodologically aligned reference study.

### 2.5 Blood collection and plasma sample preparation for metabolomic analysis

Following the 8‐week experimental period, blood samples were collected from all 12 mice at the post‐exercise intervention time point. For this, mice were anaesthetized with Avertin (2,2,2‐tribromoethanol; Sigma‐Aldrich, St. Louis, MO, USA) administered intraperitoneally at 200 mg/kg (equivalent to 2 mg per 10 g body weight), a dose commonly used to achieve short‐term surgical anaesthesia. Adequate depth of anaesthesia was confirmed by the absence of pedal withdrawal and corneal reflexes. Following confirmation of deep anaesthesia, animals were euthanized by cervical dislocation as a secondary method in accordance with approved institutional and national guidelines. Whole blood was collected from the left ventricle using sterile syringes and transferred into 500‐µL K_2_EDTA (lavender‐top) micro‐tubes (BD, Mississauga, ON, Canada). Samples were centrifuged at 1600 *g* for 15 min, and the plasma supernatant was extracted and preserved at −80°C until analysis. For metabolomic sample preparation, 50 µL of plasma was combined with 100 µL of extraction solution containing 95 µL liquid chromatography–mass spectrometry (LC–MS) grade acetonitrile (ACN) and 5 µL isotope internal standards (caffeine‐d_3_ (1‐methyl‐d_3_), 99%; Sigma‐Aldrich) at a 1:2 (v/v) ratio. The 150‐µL mixture was vortexed for 1 min and centrifuged at 16,000 × *g* at 4°C for 10 min to achieve protein precipitation and metabolite extraction. The supernatant containing polar metabolic compounds was transferred to LC–MS Autosampler vials for LC–MS/MS analysis.

### 2.6 LC–MS/MS analytical parameters for untargeted metabolomics

Untargeted metabolomics analysis was conducted using UHPLC (Thermo Fisher Scientific, Sunnyvale, CA, USA) coupled with a TripleTOF 5600+ mass spectrometry system (AB Sciex, Foster City, CA, USA). Chromatographic separation was achieved using a Hypersil GOLD aQ C18 column (100 mm × 2.1 mm; particle size 1.9 µm) (Thermo Scientific) maintained at 45°C. The mobile phase consisted of Solvent A [LC–MS grade water (H_2_O) (J.T. Baker, Phillipsburg, NJ, USA) containing 0.1% formic acid (CH_2_O_2_) (Sigma‐Aldrich)] and Solvent B [LC–MS grade acetonitrile (CH_3_CN) (J.T. Baker) containing 0.1% formic acid]. The UHPLC gradient programme was optimized as follows: 0.0–1.0 min, 5% Solvent B; 1.0–9.0 min, 5–45% Solvent B; 9.0–12.0 min, 45–90% Solvent B; 12.0–16.0 min, 90% Solvent B, followed by a 4‐min equilibration period with 5% Solvent B. The flow rate was maintained at 0.4 mL/min throughout the analysis. Sample injection volume was set to 3 µL using an autosampler maintained at 4°C. Mass spectrometric detection was performed using the following parameters: capillary voltage of 5.5 kV, drying and sheath gas temperature of 500°C, and positive electrospray ionization (+ESI) mode. Mass‐to‐charge ratio (m/z) detection range was established between 50.0 and 1000.0 with a resolution of ≥35,000 based on full width at half maximum. All plasma samples were analysed in triplicate using randomized injection sequences to ensure analytical reproducibility and statistical reliability. Injection needle cleaning was performed between samples using 150 µL of 100% acetonitrile to prevent cross‐contamination. Metabolite identification was based on mass‐to‐charge ratio (m/z), retention time (RT), and metabolic peak intensity (MPI), with real‐time spectral monitoring conducted using base peak chromatogram (BPC) and total ion chromatogram (TIC) during LC–MS acquisition.

### 2.7 Metabolomics data processing and bioinformatics analysis

Raw metabolomic feature data from the TripleTOF 5600+ mass spectrometry system were obtained using MarkerView software (AB SCIEX, Foster City, CA, USA) to generate *m*/*z*, RT, and MPI values. The raw LC–MS spectral data in ‘.wiff’ format were converted to ‘.mzXML’ format utilizing MSConvert (Adusumilli & Mallick, 2017), a command‐line utility for mass spectrometry file conversion. The resulting mzXML files were subjected to comprehensive analysis using apLCMS (R‐driven package, v. 3.4.3), which implements sophisticated computational algorithms for metabolic feature detection, quantification and high‐precision feature alignment (Yu et al., 2009), yielding datasets comprising *m*/*z*, RT and MPI parameters. Subsequently, apLCMS output underwent rigorous evaluation and correction using xMSanalyzer (R‐driven package, ver. 3.4.3) to ensure seamless dataset integration and comprehensive quality control through sample assessment, feature consistency validation and batch‐effect correction for the triplicate‐analysed plasma samples (Uppal et al., 2013). The refined datasets from xMSanalyzer were processed for comprehensive metabolite annotation using xMSannotator (R‐driven package, ver. 3.2.5) (Uppal et al., 2017), an advanced network‐based R computational framework specifically designed for automated annotation of high‐resolution metabolomics datasets. The annotated metabolites were further analysed through systematic filtering, normalization, statistical analysis and intergroup comparative analysis using MetaboAnalyst 6.0 (Pang et al., 2024), a comprehensive web‐based analytical platform designed for integrated metabolomics data processing, analysis and biological interpretation. Statistical significance between groups was assessed using an independent samples Student's *t*‐test. To account for multiple testing in the untargeted metabolomics dataset, false discovery rate (FDR) correction was performed using the Benjamini–Hochberg method. Both raw *P*‐values and FDR‐adjusted *P*‐values were evaluated, and the full list of metabolites with corresponding FDR‐adjusted *P*‐values is provided in Table S1. Receiver operating characteristic (ROC) curve analysis was also performed to assess the discriminative performance of key metabolites.

### 2.8 Computational metabolomics workflow and feature selection

Metabolic raw data obtained from the untargeted metabolomics process were initially processed using apLCMS to extract fundamental metabolic information. The processed data were subsequently analysed using xMSanalyzer, which generated a comprehensive dataset containing 13,793 metabolic features with corresponding *m*/*z*, RT and MPI. To eliminate excessive zero‐value intensities that could compromise data quality, a filtering protocol was applied whereby metabolic features containing more than 30% (0.3 ratio) zero values across all samples from both CG and AEG were systematically excluded from the comparative analysis dataset; this filtering process resulted in a refined dataset comprising 10,896 metabolic features. Then, variable importance in projection (VIP) score analysis was performed on the dataset of 10,896 metabolic features using the MetaboAnalyst 6.0 platform. We selected metabolic features using a VIP score threshold of ≥1.0, which represents an established statistical criterion recognized for indicating meaningful discriminatory power in metabolomics research (Akarachantachote et al., 2014). This selection criterion yielded 867 metabolic features with significant discriminatory potential. To perform the metabolite identification and annotation, the 867 selected metabolic profiles were processed through xMSannotator for KEGG identifier (known as KEGG ID) and metabolite nomenclature; for this, error tolerance of ±10 ppm (equivalent to ±0.001%) was applied to *m*/*z* values to minimize the inclusion of irrelevant annotated metabolic features in the final dataset. Following this annotation and refinement process, a final dataset of 433 metabolic profiles was obtained and utilized for all subsequent analytical processes, statistical calculations and figure generation presented in the results section.

### 2.9 Statistical analysis and power calculation

For univariate statistical analysis, two‐group comparisons were performed using the two‐sample *t*‐test module in MetaboAnalyst 6.0. All analyses were conducted in the unpaired format with unequal group variance settings. Statistical significance was determined using a threshold of *P* < 0.05, and *P*‐values were generated to identify differentially abundant metabolites. To minimize systematic variation and improve comparability across samples in univariate statistical analysis, metabolomics raw data were preprocessed prior to multivariate analysis of the metabolomic big dataset. Data normalization was performed using median normalization, which adjusts each sample by the median value of its detected metabolites to correct for sample‐specific biases. Subsequently, range scaling was used to mean‐centre and scale each metabolite intensity by its range, thereby ensuring equal weight of all metabolites regardless of their absolute concentration levels. These preprocessing steps were implemented using the built‐in functions prior to MetaboAnalyst 6.0 analysis in order to achieve reliable outcomes in subsequent analyses.

We also performed a two‐sample Welch's *t*‐test‐based power analysis using Python (version 3.11.9) with the *statsmodels.stats.power.TTestIndPower* module to justify the chosen sample size. The mean effect size (Cohen's *d* = 0.722) observed between CG and AEG reflects a moderate‐to‐large magnitude of change, implying that exercise‐induced metabolic shifts are substantial enough to have potential biological relevance beyond statistical significance. In our study, rather than relying solely on calculated power to justify the sample size, we frame this untargeted metabolomics study as a discovery‐phase investigation prioritizing biomarker identification and pathway‐level signal stability. In high‐dimensional settings where the number of features (metabolites) far exceeds the number of animals, univariate power is not the primary design criterion. Therefore, the robustness of findings was reinforced by multiple independent analytical approaches (partial least squares discriminant analysis (PLS‐DA), volcano plot, hierarchical clustering, pathway analysis, ROC curves), all of which consistently identified the up‐ and downregulation of HBP‐associated metabolites.

## 3 RESULTS

### 3.1 Metabolic discrimination between CG and AEG

**FIGURE 1:**
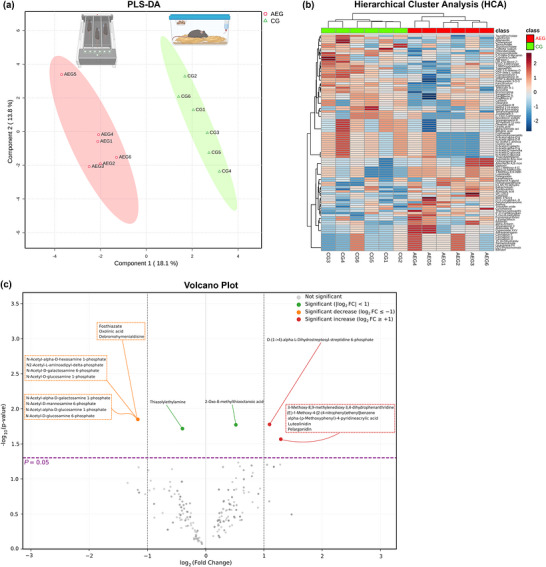
Metabolic discrimination and metabolomic profiling between groups and significantly identified metabolites. (a) PLS‐DA score plot illustrating clear metabolic separation between CG (green triangles, *n* = 6) and AEG (pink circles, *n* = 6) with Components 1 and 2 explaining 18.1% and 13.8% of the total variance, respectively. Ellipses represent 95% confidence intervals (green: CG; red: AEG). (b) Hierarchical cluster analysis heatmap displaying distinct metabolite abundance profiles between groups. Colour intensity reflects normalized metabolite intensity (red: high; blue: low; scale: −2 to +2). (c) Volcano plot of differential metabolite expression (AEG/Con) depicting log_2_ fold change (FC) (*x*‐axis) against −log_10_(*P*‐value) (*y*‐axis). Metabolites appearing on the left side of the plot (negative log_2_ FC) represent those decreased in AEG relative to CG, whereas those on the right side (positive log_2_ FC) represent metabolites increased in AEG. The horizontal dashed line indicates the significance threshold (*P* = 0.05). Orange circles represent metabolites with a significant decrease in the exercise group (log_2_ FC ≤ −1), red circles indicate metabolites with significant increase (log_2_ FC ≥ +1), green circles denote statistically significant metabolites with smaller fold changes (|log_2_ FC| < 1), and grey circles represent non‐significant metabolites.

To investigate the metabolic differences between CG and AEG, we performed partial least squares discriminant analysis (PLS‐DA) on the metabolomics dataset. Figure 1a shows the PLS‐DA score plot where Components 1 and 2 explained 18.1 and 13.8% of the total variance, respectively, and the evaluation was based on two key metrics, *R* ^2^ (explained variance) and *Q* ^2^ (cross‐validation predictive ability). The two‐component PLS‐DA model achieved comprehensive validation metrics demonstrating its reliability and biological relevance. The model attained a classification accuracy of 1.000 (100%), indicating that 100% of samples could be correctly assigned to their respective groups based on their metabolic profiles. The *R* ^2^ value of 0.981 demonstrated that the model explained 98.1% of the variance in the training dataset, while the *Q* ^2^ value of 0.673 confirmed robust predictive ability under cross‐validation conditions. To evaluate potential overfitting, we calculated the difference between *R* ^2^ and *Q* ^2^ values for each model configuration. The analysis revealed acceptable overfitting indices for the two‐component model, with an *R* ^2^–*Q* ^2^ difference of 0.308, which remains within the commonly accepted threshold of <0.35 for metabolomics PLS‐DA models. These results indicate that the model demonstrates adequate predictive performance without substantial overfitting and successfully discriminates metabolic profiles between CG and AEG.

### 3.2 Hierarchical cluster analysis of metabolic profiles

As a means of validating metabolic discrimination and visualizing overall metabolite expression patterns between groups, we performed hierarchical cluster analysis (HCA) on the metabolomics dataset. Figure 1b presents the comprehensive heatmap showing both sample clustering (top dendrogram) and metabolite clustering (left dendrogram) between CG and AEG. Sample clustering demonstrated a clear tendency for group separation, which corroborates the PLS‐DA findings (Figure 1a). Notably, *N*‐Acetyl compound clusters showed consistently elevated expression in the CG compared to AEG; therefore, systematic metabolic reprogramming likely occurred following VAAE.

### 3.3 Differential metabolite expression analysis using volcano plot

To identify specific metabolites contributing to the group discrimination observed in the PLS‐DA and HCA, we performed differential expression analysis and visualized the results using a volcano plot. Figure 1c presents the volcano plot displaying the relationship between statistical significance (−log_10_(*P*‐value)) and biological effect size (log_2_ fold change) for all detected metabolites. The most prominent finding from the volcano plot analysis was the downregulation of *N*‐acetyl compounds following VAAE in AEG. A total of eight authentic *N*‐acetyl compounds were identified as significantly decreased in AEG compared with CG (*P* = 0.0139). These metabolites showed a fold change of 0.445 (AEG/CG), indicating an approximately 55.5% reduction in AEG relative to CG. The coordinated reduction of *N*‐acetyl compounds by 55.5% relative to CG levels represented the most pronounced and consistent metabolic change observed following aerobic exercise intervention. Collectively, the convergent findings across the volcano plot, PLS‐DA and HCA provided consistent evidence for exercise‐induced metabolic changes and established quantitative parameters for the observed group differences. To further address multiple testing, Benjamini–Hochberg FDR correction was applied to all detected features. Although several metabolites reached nominal significance based on raw *P*‐values, no individual metabolite remained significant after FDR correction (FDR < 0.05). FDR‐adjusted *P*‐values are provided in Table S1.

### 3.4 Metabolic pathway analysis and statistical significance assessment

**FIGURE 2:**
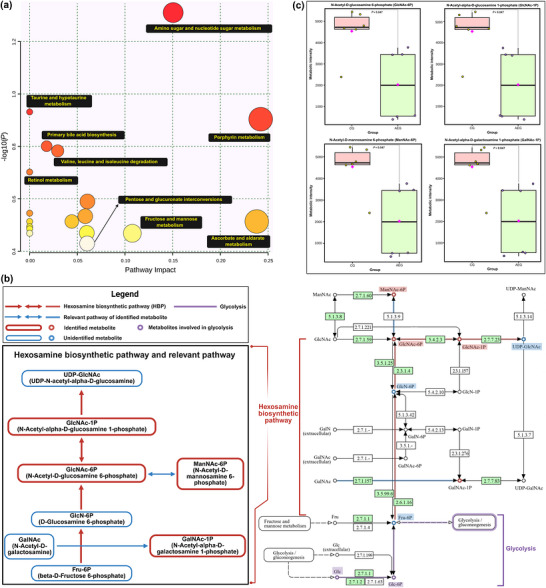
Pathway enrichment analysis, hexosamine biosynthetic pathway visualization, and metabolic intensity of key metabolites. (a) Scatter plot of metabolite set enrichment analysis depicting pathway impact (*x*‐axis) against −log_10_(*P*‐value) (*y*‐axis). Circle size and colour intensity reflect pathway impact and statistical significance, respectively. Amino sugar and nucleotide sugar metabolism was the most significantly enriched pathway, followed by taurine and hypotaurine metabolism, primary bile acid biosynthesis, and porphyrin metabolism. (b) Schematic representation of the HBP and connected metabolic networks. Red‐outlined boxes indicate identified metabolites, and blue‐outlined boxes represent undetected metabolites. The KEGG‐based pathway map (right) illustrates broader connections between hexosamine biosynthesis and glycolysis. (c) Box plots of four HBP‐related metabolites (GlcNAc‐6P, GlcNAc‐1P, ManNAc‐6P and GalNAc‐1P) comparing metabolic intensity between CG and AEG. All four metabolites were significantly lower in AEG (*P* = 0.047). Pink and green boxes represent the control (CG) and aerobic exercise group (AEG), respectively. Horizontal lines and pink diamonds indicate median and mean values. Statistical significance was determined at *P* < 0.05.

Pathway enrichment analysis was performed using the identical metabolomics dataset employed for Figure 1 in order to identify the metabolic pathways most affected by VAAE. Figure 2a presents the pathway analysis results as a scatter plot by displaying the relationship between pathway impact score (*x*‐axis) and statistical significance (−log_10_ *P*‐value at *y*‐axis) for all enriched metabolic pathways. The pathway analysis identified nine metabolic pathways with varying degrees of pathway impact scores and statistical significance. Among all analysed pathways, amino sugar and nucleotide sugar (ASNS) metabolism was the only pathway that achieved statistical significance (*P* = 0.049, FDR = 0.360) with a pathway impact score of 0.150 (Figure 2a and Table 1). ASNS metabolism encompasses the HBP, as confirmed by KEGG database analysis, and includes the four key *N*‐acetyl metabolites identified in our study (Figure 2b): *N*‐acetyl‐d‐glucosamine 6‐phosphate (GlcNAc‐6P), *N*‐acetyl‐α‐d‐glucosamine 1‐phosphate (GlcNAc‐1P), *N*‐acetyl‐d‐mannosamine 6‐phosphate (ManNAc‐6P), and *N*‐acetyl‐α‐d‐galactosamine 1‐phosphate (GalNAc‐1P). ASNS metabolism demonstrated a match status of 5/42, indicating that five out of 42 metabolites in this pathway were identified as significantly altered in our dataset (Table 1). Additionally, ASNS metabolism contained the aforementioned four key *N*‐acetyl metabolites that were previously identified as discriminatory compounds in the volcano plot and HCA. These metabolites corresponded to the downregulation observed in AEG (reduced by 55.5% compared to CG, *P* = 0.0139) as demonstrated in the volcano plot analysis (Figure 1c).

**TABLE 1:** Summary of identified pathways and statistical values.

Identified pathway name	Match status	*P*	FDR	Impact score
Amino sugar and nucleotide sugar metabolism	5/42	0.049	0.360	0.150
Taurine and hypotaurine metabolism	1/8	0.117	0.360	0.000
Porphyrin metabolism	4/31	0.125	0.360	0.240
Primary bile acid biosynthesis	2/46	0.158	0.360	0.020
Valine, leucine and isoleucine degradation	1/40	0.165	0.360	0.030
Retinol metabolism	1/17	0.199	0.360	0.000
Ascorbate and aldarate metabolism	1/9	0.307	0.360	0.240
Pentose phosphate pathway	1/23	0.307	0.360	0.040
Fructose and mannose metabolism	1/20	0.340	0.360	0.110

*Note*: Match status indicates the number of matched metabolites relative to the total number of metabolites in each pathway (hits/total). False discovery rate (FDR) was calculated to account for multiple comparisons. Pathway impact scores were calculated based on topology analysis, with scores greater than zero considered indicative of metabolic perturbation within the respective pathway.

Nominal *P*‐values are presented, with statistical significance set at *P* < 0.05.

### 3.5 KEGG‐based metabolic visualization of hexosamine biosynthetic pathway

For validation of the metabolic positioning of the four significantly identified *N*‐acetyl compounds from the previous pathway analysis (Figure 2a and Table 1), an integrated metabolic network diagram was developed based on KEGG pathways (Figure 2b). The analysis showed that the HBP is closely connected to glycolysis. Although glucose (Glc) and glucose‐6‐phosphate (Glc‐6P) were not detected as significantly altered metabolites in our study, KEGG pathway mapping illustrated their connection to the HBP network. The diagram showed that glucose can be metabolically channeled into two distinct pathways: the HBP route leading to UDP‐GlcNAc formation (indicated by red pathway lines) or the glycolytic cascade for energy generation (indicated by purple pathway lines). Within the core HBP framework, two key metabolic intermediates (GlcNAc‐6P and GlcNAc‐1P) were identified as direct components of the core HBP. These compounds, highlighted as identified metabolites (red boxes) in the pathway diagram, represent sequential intermediates in the glucose‐to‐UDP‐GlcNAc conversion pathway. ManNAc‐6P and GalNAc‐1P were identified as significantly altered compounds; however, the KEGG mapping showed their positioning as auxiliary metabolites within the broader ASNS metabolism network rather than as direct intermediates within the classical HBP route. UDP‐GlcNAc, the terminal product of the HBP, was not detected as a significantly altered metabolite in our analysis.

### 3.6 ROC curve analysis of key metabolic features

**FIGURE 3:**
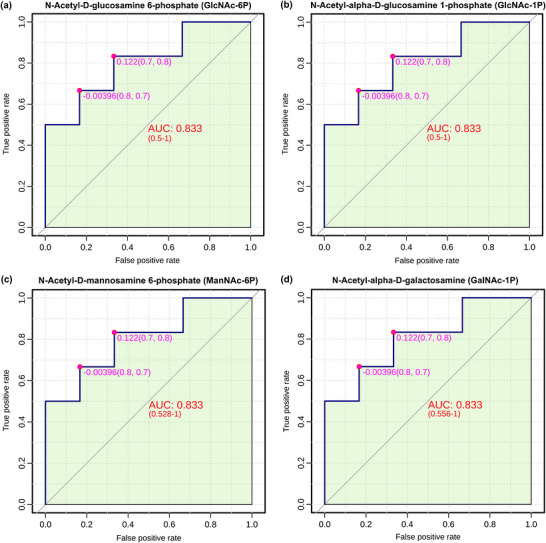
Receiver operating characteristic (ROC) curve analysis for HBP‐related metabolites. ROC curves are presented for (a) GlcNAc‐6P, (b) GlcNAc‐1P, (c) ManNAc‐6P and (d) GalNAc‐1P. All four metabolites demonstrated identical discriminative performance with an AUC of 0.833, with 95% confidence intervals of 0.500–1.000 for GlcNAc‐6P and GlcNAc‐1P, 0.528–1.000 for ManNAc‐6P, and 0.556–1.000 for GalNAc‐1P. The *x*‐axis represents the false positive rate (1 − specificity), and the *y*‐axis represents the true positive rate (sensitivity). Pink dots indicate two optimal cutoff points for each metabolite: a sensitivity‐prioritized cutoff at −0.00396 (sensitivity = 0.8, specificity = 0.7) and a specificity‐prioritized cutoff at 0.122 (sensitivity = 0.7, specificity = 0.8). The grey diagonal line represents chance‐level discrimination (AUC = 0.5). The consistent AUC values across all four metabolites suggest their collective utility as candidate biomarkers of exercise‐induced modulation of the hexosamine biosynthetic pathway.

ROC curve analysis was performed to evaluate the discriminatory performance of the four identified *N*‐acetyl compounds (Figure 3). All four metabolites demonstrated identical area under the curve (AUC) values of 0.833, indicating strong classification performance. GlcNAc‐6P and GlcNAc‐1P (Figure 3a,b) representing core intermediates within the HBP showed AUC values of 0.833 with confidence intervals of 0.5–1. ManNAc‐6P and GalNAc‐1P (Figure 3c,d) positioned as auxiliary metabolites in ASNS exhibited identical AUC values of 0.833 with confidence intervals of 0.528–1 and 0.556–1, respectively. The optimal cutoff points were consistent across all four metabolites, with two distinct thresholds identified: −0.00396 corresponding to sensitivity and specificity coordinates of (0.8, 0.7), and 0.122 corresponding to coordinates of (0.7, 0.8). The ROC curves demonstrated that both the core HBP intermediates (GlcNAc‐6P and GlcNAc‐1P) and the auxiliary metabolites (ManNAc‐6P and GalNAc‐1P) exhibited equivalent discriminatory capacity. All metabolites showed performance substantially above the diagonal reference line representing random classification (AUC = 0.5).

### 3.7 Exercise‐induced suppression of HBP‐associated *N*‐acetyl metabolites

Comparative analysis of metabolic intensities between CG and AEG revealed a significant difference across all four *N*‐acetyl compounds (Figure 2c). The core HBP intermediates, GlcNAc‐6P and GlcNAc‐1P, showed significant reductions in AEG compared to CG (*P* = 0.047 for both metabolites). The auxiliary amino sugar metabolites, ManNAc‐6P and GalNAc‐1P, exhibited identical patterns of significant downregulation following VAAE (*P* = 0.047 for both metabolites). All four metabolites demonstrated approximately two‐fold or greater reductions in mean intensities in AEG, with consistent *P*‐values of 0.047 across all comparisons.

## 4 DISCUSSION

The present study demonstrates that an 8‐week VAAE intervention significantly modulates the HBP that is known to be closely connected to glucose metabolism. The exercise intensity applied to AEG (approximately 65.72% of maximal ${\dot{V}}_{O_{2\max}}$, equivalent to 69.1 mL/kg/min) represents a physiologically demanding intervention that aligns with established principles of exercise‐induced metabolic substrate utilization. According to previous exercise physiology literature, aerobic exercise at intensities exceeding 65% of maximal ${\dot{V}}_{O_{2\max}}$ preferentially activates carbohydrate metabolism over fat oxidation (Romijn et al., 1993; van Loon et al., 2001). This metabolic shift is particularly relevant given that the estimated exercise intensity in our study (65.72% ${\dot{V}}_{O_{2\max}}$) positions the intervention at the threshold where carbohydrate oxidation becomes the predominant energy source. This finding is consistent with the exercise‐intensity‐dependent substrate utilization patterns, where fat oxidation predominates at lower intensities (25–40% ${\dot{V}}_{O_{2\max}}$) while carbohydrate metabolism becomes increasingly important at higher intensities (75–85% ${\dot{V}}_{O_{2\max}}$) (Hargreaves & Spriet, 2020; Romijn et al., 1993; van Loon et al., 2001).

The all‐out aerobic exercise intensity applied in our study likely created metabolic conditions favouring enhanced glucose utilization through glycolysis, thereby reducing glucose flux toward alternative pathways such as the HBP. This metabolic shift reflects the fundamental principle of glucose partitioning in cellular metabolism. Given that glycolysis accounts for 95–98% of cellular glucose utilization while the HBP accounts for only 2–5% (Hawkins et al., 1997; Marshall et al., 1991; Traxinger & Marshall, 1991), vigorous aerobic exercise at 65.72% of maximal ${\dot{V}}_{O_{2\max}}$ is hypothesized to further augment glycolytic demand, thereby competitively reducing glucose availability for the HBP. This enhanced glycolysis promotes glucose (Glc) utilization and oxidation, potentially reducing glucose allocation to the HBP (Figure 2c). Therefore, the enhanced glycolytic demand during high‐intensity aerobic exercise creates a competitive metabolic environment where glucose preferentially flows toward energy production rather than the HBP, which supports our observed downregulation of HBP‐associated metabolites identified in AEG.

The most significant finding of this study is the downregulation of HBP‐associated metabolites following aerobic exercise intervention, as comprehensively demonstrated through multiple analytical approaches. The PLS‐DA score plot (Figure 1a) successfully discriminated between CG and AEG, achieving a classification accuracy of 100% (*R* ^2^ = 0.981, *Q* ^2^ = 0.673). HCA confirmed metabolic discrimination through clear group separation, with *N*‐acetyl compound clusters showing consistently reduced expression in AEG that reflects systematic metabolic reprogramming following VAAE (Figure 1b). The volcano plot provided quantitative evidence for the exercise‐induced metabolic changes; it demonstrates that *N*‐acetyl compounds were significantly downregulated in AEG with both statistical significance (*P* = 0.0139) and substantial biological effect (fold change = 0.445) (Figure 1c). In AEG, these metabolites were reduced to approximately 44.5% of CG levels, corresponding to an ∼55.5% decrease, which suggests a substantial suppression of HBP‐related metabolic activity following aerobic exercise. The major HBP intermediates, GlcNAc‐6P and GlcNAc‐1P, along with auxiliary amino sugar metabolites, ManNAc‐6P and GalNAc‐1P, demonstrated consistent and significant reductions (*P* = 0.047 for all metabolites) with approximately two‐fold decreases in the AEG compared to CG (Figure 2c). This systematic suppression of HBP activity represents a novel mechanistic insight into exercise‐induced metabolic adaptations.

As described above, only 2–5% of glucose enters the HBP while 95–98% goes to glycolysis (Hawkins et al., 1997; Marshall et al., 1991; Traxinger & Marshall, 1991); the HBP produces UDP‐*N*‐acetylglucosamine (UDP‐GlcNAc) that serves as a substrate for protein *O*‐GlcNAcylation, a post‐translational modification implicated in insulin resistance and metabolic dysfunction (Hart et al., 2007). Although UDP‐GlcNAc was not detected as a significantly altered metabolite in our analysis, the observed downregulation of HBP intermediates (GlcNAc‐6P and GlcNAc‐1P) suggests that all‐out aerobic exercise effectively redirects glucose flux toward glycolysis, potentially through mass action effects and enhanced glycolytic enzyme activity, which indicates the significant metabolic pathway reorganization. Pathway enrichment analysis further characterized these metabolic changes (Figure 2a), detecting nine altered metabolic routes. Among these, ASNS metabolism was the sole significant pathway (*P* = 0.0489; FDR = 0.36; impact score 0.15) (Table 1), with 5/42 matched metabolites reflecting HBP involvement. KEGG‐based visualization (Figure 2b) positioned the *N*‐acetyl compounds within the HBP's core framework with GlcNAc‐6P and GlcNAc‐1P as sequential UDP‐GlcNAc precursors and ManNAc‐6P and GalNAc‐1P as ancillary amino sugar metabolites.

The elevated HBP intermediates observed in the CG may reflect a metabolic signature of physical inactivity and contribute to the systemic dysregulation associated with a sedentary lifestyle. Under conditions of restricted physical activity and ad libitum access to a carbohydrate‐rich diet (44.2%), the CG effectively modelled a sedentary, hypercaloric state. This metabolic environment likely intensified glucose flux through the HBP, which reflects the systemic consequences of energy surplus and physical inactivity. Previous studies have reported that sedentary behaviour has been associated with greater susceptibility to type 2 diabetes and metabolic dysfunction (Hu et al., 2003). Furthermore, excessive intake of carbohydrate‐rich foods has been shown to trigger hyperglycaemia and elevated glucagon levels (Garg et al., 1992). Consistent with these observations, our experimental design intentionally created glucose‐rich conditions that exceeded glycolytic capacity, thereby directing surplus glucose into the HBP via mass action. This interpretation is further supported by evidence demonstrating that when glucose levels surpass glycolytic capacity, the resulting increase in HBP flux induces compensatory decreases in glucose transport and insulin action (Marshall et al., 1991). The significantly elevated HBP intermediates in the CG suggest that the combination of sedentary conditions and high carbohydrate availability fostered a metabolic environment favourable for both HBP activation and the potential development of insulin resistance. Consequently, the exercise‐induced suppression of HBP intermediates observed in the current study identifies a plausible mechanism by which high‐intensity aerobic exercise may improve glucose homeostasis and enhance insulin sensitivity.

However, these findings must be interpreted with consideration for tissue‐ and cell‐type specificity. While our plasma metabolomic data show a systemic reduction in HBP‐associated intermediates after VAAE, plasma levels do not always mirror metabolic flux within specific tissues, such as skeletal muscle. Recent evidence suggests that HBP activation in endothelial cells actually promotes adaptive angiogenic responses. Specifically, glucosamine‐induced HBP activation has been shown to enhance angiogenesis and perfusion in ischaemic muscle by promoting oxidative phosphorylation, activating the serine biosynthesis pathway to support nucleotide synthesis and redox balance, and engaging ATF4‐mediated signalling that drives endothelial cell survival and angiogenic gene expression, notably without increasing glycolysis (Alhusban et al., 2024). Furthermore, exercise in patients with peripheral arterial disease is linked to increased endothelial *O*‐GlcNAcylation, which implies that localized HBP activation contributes to vascular remodelling (Alhusban et al., 2024). These insights highlight the divergent regulation of the HBP across different biological contexts. Therefore, while our data indicate a systemic shift of glucose away from the HBP under high‐intensity aerobic conditions, localized HBP signalling in populations like endothelial cells may still serve as an adaptive driver for exercise‐induced remodelling. Future research using tissue‐specific metabolic flux analysis is necessary to clarify the tissue‐ and cell‐type‐specific regulation of the HBP.

The clinical implications of these findings extend to metabolic disease prevention and management. ROC curve analysis (Figure 3) demonstrated strong discriminatory performance for all four identified *N*‐acetyl compounds, with identical AUC values of 0.833 for GlcNAc‐6P, GlcNAc‐1P, ManNAc‐6P and GalNAc‐1P. These consistent AUC values indicate the potential utility of these metabolites as biomarkers for exercise‐induced metabolic adaptations. Elevated HBP activity and increased protein *O*‐GlcNAcylation have been implicated in various metabolic complications, including insulin resistance in pancreatic β‐cells (Akimoto et al., 2007), diabetes‐related complications in corneal tissue (Akimoto et al., 2003) and cardiovascular complications associated with diabetes (Buse, 2006).

In conclusion, the exercise intensity employed in this study (approximately 65.72% ${\dot{V}}_{O_{2\max}}$) is clinically relevant for suppressing HBP activity, yet may present adherence challenges for sedentary individuals or those at risk of metabolic disorders such as type 2 diabetes. While this intensity could theoretically be incorporated into evidence‐based exercise prescription protocols, it is important to acknowledge that 65.72% ${\dot{V}}_{O_{2\max}}$ represents a relatively heavy exercise intensity for sedentary individuals at risk of developing type‐2 diabetes or those already diagnosed with metabolic syndrome. In practical terms, achieving and maintaining this exercise intensity may present significant physiological and adherence challenges for deconditioned populations who typically exhibit lower baseline fitness levels and reduced exercise tolerance. However, the metabolic rationale for pursuing higher exercise intensities becomes evident when considering established substrate utilization patterns in glycolysis. Previous research has demonstrated that carbohydrate metabolism becomes increasingly important at higher intensities (75–85% ${\dot{V}}_{O_{2\max}}$) (Hargreaves & Spriet, 2020; Romijn et al., 1993; van Loon et al., 2001), indicating that enhanced glucose flux through glycolysis occurs in a dose‐dependent manner with exercise intensity. Therefore, despite the practical challenges associated with achieving 65.72% ${\dot{V}}_{O_{2\max}}$ in clinical populations, the importance of progressing toward higher exercise intensities to effectively suppress HBP activation and maximize glucose utilization cannot be understated. This progressive approach to higher‐intensity exercise is particularly crucial for weight management individuals, metabolic disease patients, and those with diabetes‐related symptoms. Even without fully reaching the 65.72% ${\dot{V}}_{O_{2\max}}$ target, the gradual increase in aerobic exercise intensity at personally appropriate levels represents the core component of exercise intervention and serves as a pivotal strategy for attenuating HBP activation. From the standpoint of exercise metabolism, it is crucial to recognize that individuals with metabolic risk factors should prioritize pursuing gradually higher aerobic exercise intensities regardless of initial success or failure, as this approach can further enhance carbohydrate and glucose metabolism.

Nevertheless, several limitations should be noted. First, future animal studies should incorporate direct ${\dot{V}}_{O_{2\max}}$ measurement to more precisely delineate exercise‐intensity‐dependent metabolic pathway dynamics. Second, validation in human subjects will be necessary to determine whether the metabolic trends observed in the present study are recapitulated in a clinical context.

## AUTHOR CONTRIBUTIONS

Gyumin Kang and Young Jun Kim conceived and designed the study. Gyumin Kang and Young Jun Kim acquired, analysed, and interpreted the data. Gyumin Kang, Jae Kyeom Kim, Eui‐Cheol Shin, and Young Jun Kim drafted the manuscript and revised it critically for important intellectual content. All authors have read and approved the final version of this manuscript and agree to be accountable for all aspects of the work in ensuring that questions related to the accuracy or integrity of any part of the work are appropriately investigated and resolved. All persons designated as authors qualify for authorship, and all those who qualify for authorship are listed.

## CONFLICT OF INTEREST

None declared.

## Supporting information




**Supplementary Table 1**. Differential metabolite analysis used for volcano plot visualization (401 metabolites).

## Data Availability

The data that support the findings of this study are available from the corresponding author upon reasonable request. Certain aspects of the dataset may be subject to intellectual property considerations or potential commercial applications, and access will therefore be provided in accordance with institutional policies and applicable regulations.
