# Supplementary material for: V˙O2max‐based all‐out aerobic exercise attenuates hexosamine biosynthetic pathway activity: Metabolomic insights in mice model
Source: Exp Physiol. 2026 Jun 11;111(7):3253–66. doi: 10.1113/EP093528 (PMC13327345; doi:10.1113/EP093528)
Supplement: Supplementary file 1 — Supplementary Table 1. Differential metabolite analysis used for volcano plot visualization (401 metabolites). [file EPH-111-3253-s001.docx]

**Supplementary Table 1.** Differential metabolite analysis used for volcano plot visualization (401 metabolites)

| **Metabolite** | **log_2_FC** | **p-value** | **FDR (BH)** | **-log_10_(p)** | **Significant (FDR < 0.05)** |
| --- | --- | --- | --- | --- | --- |
| GDP-mannose;GDP-D-mannose;GDP-alpha-D-mannose | 0.590 | 0.345 | 0.509 | 0.462 | No |
| Glycogen | 0.231 | 0.387 | 0.509 | 0.412 | No |
| D-Gluconic acid;D-Gluconate;D-gluco-Hexonic acid | 0.394 | 0.393 | 0.509 | 0.406 | No |
| N-Acetyl-D-glucosamine 6-phosphate | -1.167 | 0.0139 | 0.507 | 1.856 | No |
| Quercetin;3,3',4,5,7-Pentahydroxyflavone;3,5,7,3',4'-Pentahydroxyflavone | 1.170 | 0.0673 | 0.509 | 1.172 | No |
| GDP-glucose;GDP-D-glucose;GDP-alpha-D-glucose | 0.590 | 0.345 | 0.509 | 0.462 | No |
| (-)-Menthol;L-Menthol | -0.006 | 0.991 | 0.991 | 0.004 | No |
| Bilirubin | 0.207 | 0.396 | 0.509 | 0.402 | No |
| Biliverdin;Biliverdin IX alpha | 0.489 | 0.143 | 0.509 | 0.843 | No |
| D-Mannonate | 0.394 | 0.393 | 0.509 | 0.406 | No |
| (+)-Neomenthol | -0.006 | 0.991 | 0.991 | 0.004 | No |
| L-Idonate | 0.394 | 0.393 | 0.509 | 0.406 | No |
| CDP-ribitol;CDP-L-ribitol;CDP 5-ester with D-ribitol | -0.131 | 0.78 | 0.8 | 0.108 | No |
| L-Gulonate;L-Gulonic acid;Gulonate;Gulonic acid | 0.394 | 0.393 | 0.509 | 0.406 | No |
| D-Altronate | 0.394 | 0.393 | 0.509 | 0.406 | No |
| Coenzyme F420;Oxidized coenzyme F420 | 0.223 | 0.646 | 0.704 | 0.190 | No |
| D-Galactonate;D-Galactonic acid | 0.394 | 0.393 | 0.509 | 0.406 | No |
| Gentamicin C1a | 0.238 | 0.354 | 0.509 | 0.451 | No |
| Protoporphyrinogen IX | 0.152 | 0.484 | 0.573 | 0.315 | No |
| 1D-myo-Inositol 1,3,4,5,6-pentakisphosphate;D-myo-Inositol 1,3,4,5,6-pentakisphosphate;Inositol 1,3,4,5,6-pentakisphosphate | 0.252 | 0.415 | 0.521 | 0.382 | No |
| Xanthan;Xanthene | -0.420 | 0.253 | 0.509 | 0.597 | No |
| GDPhexose | 0.590 | 0.345 | 0.509 | 0.462 | No |
| Stachyose | 0.231 | 0.387 | 0.509 | 0.412 | No |
| Undecaprenol | -0.881 | 0.357 | 0.509 | 0.448 | No |
| Cellotetraose | 0.231 | 0.387 | 0.509 | 0.412 | No |
| Maltotetraose | 0.231 | 0.387 | 0.509 | 0.412 | No |
| GDP-L-galactose | 0.590 | 0.345 | 0.509 | 0.462 | No |
| **Metabolite** | **log_2_FC** | **p-value** | **FDR (BH)** | **-log_10_(p)** | **Significant (FDR < 0.05)** |
| (2S)-Flavan-4-ol | -0.431 | 0.327 | 0.509 | 0.485 | No |
| Retinyl palmitate;Vitamin A palmitate;all-trans-Retinyl palmitate;Retinol palmitate | -0.307 | 0.466 | 0.563 | 0.331 | No |
| Taurolithocholate;Taurolithocholic acid | -0.413 | 0.11 | 0.509 | 0.958 | No |
| Dodecanoic acid;Dodecanoate;Dodecylcarboxylate;Lauric acid | 0.227 | 0.142 | 0.509 | 0.848 | No |
| N-Acetylmethionine;N-Acetyl-L-methionine | -0.533 | 0.165 | 0.509 | 0.784 | No |
| 3-Methyleneoxindole | -0.652 | 0.53 | 0.605 | 0.276 | No |
| 8-Hydroxykaempferol | 1.170 | 0.0673 | 0.509 | 1.172 | No |
| Cypridina luciferin | -0.444 | 0.195 | 0.509 | 0.710 | No |
| N-Acetylmuramoyl-Ala;N-Acetyl-D-muramoyl-L-alanine | -0.266 | 0.349 | 0.509 | 0.457 | No |
| (+)-Bornane-2,5-dione;2,5-Diketocamphane | -0.420 | 0.253 | 0.509 | 0.597 | No |
| Diisopropyl phosphate | -0.420 | 0.253 | 0.509 | 0.597 | No |
| Zinc protoporphyrin-9;Zinc protoporphyrin | 0.317 | 0.406 | 0.515 | 0.392 | No |
| dTDP-6-deoxy-beta-L-talose;dTDP-beta-L-pneumose | 0.415 | 0.23 | 0.509 | 0.638 | No |
| 2-Carboxy-D-arabinitol | 0.394 | 0.393 | 0.509 | 0.406 | No |
| 3-Methylglutaconyl-CoA;trans-3-Methylglutaconyl-CoA;(E)-3-Methylglutaconyl-1-CoA | 0.788 | 0.0644 | 0.509 | 1.191 | No |
| dTDP-L-rhamnose;dTDP-6-deoxy-L-mannose;dTDP-6-deoxy-beta-L-mannose;dTDP-beta-L-rhamnose | 0.415 | 0.23 | 0.509 | 0.638 | No |
| 3-Methylthiopropanamine;3-Methylthiopropylamine | -0.652 | 0.53 | 0.605 | 0.276 | No |
| dTDP-L-dihydrostreptose | 0.415 | 0.23 | 0.509 | 0.638 | No |
| 11-cis-Retinyl palmitate | -0.307 | 0.466 | 0.563 | 0.331 | No |
| Gibberellin A8;2beta-Hydroxygibberellin 1 | -0.266 | 0.349 | 0.509 | 0.457 | No |
| Taurolithocholate sulfate;3alpha-Sulfatolithocholyltaurine;Taurolithocholic acid 3-sulfate | -0.432 | 0.386 | 0.509 | 0.413 | No |
| 2'-Hydroxypseudobaptigenin | 1.170 | 0.0673 | 0.509 | 1.172 | No |
| N2,N5-Dibenzoyl-L-ornithine;L-Ornithuric acid | 0.905 | 0.163 | 0.509 | 0.787 | No |
| 1-Oleoylglycerophosphocholine;1-Oleoyl-sn-glycero-3-phosphocholine | -0.200 | 0.698 | 0.737 | 0.156 | No |
| Isolychnose;3F-alpha-D-Galactosylraffinose | 0.231 | 0.387 | 0.509 | 0.412 | No |
| **Metabolite** | **log_2_FC** | **p-value** | **FDR (BH)** | **-log_10_(p)** | **Significant (FDR < 0.05)** |
| Tetrahydropteroyltri-L-glutamate;(6S)-H4PteGlu3 | -0.943 | 0.106 | 0.509 | 0.974 | No |
| Flavonol 3-O-[alpha-L-rhamnosyl-(1->6)-beta-D-glucoside];Flavonol 3-O-rutinoside | 0.214 | 0.385 | 0.509 | 0.414 | No |
| all-trans-Pentaprenyl diphosphate;Geranylfarnesyl diphosphate | 0.410 | 0.274 | 0.509 | 0.563 | No |
| N-Acetyl-D-glucosamine 1-phosphate | -1.167 | 0.0139 | 0.507 | 1.856 | No |
| N-Acetyl-D-mannosamine 6-phosphate;N-Acetylmannosamine 6-phosphate | -1.167 | 0.0139 | 0.507 | 1.856 | No |
| 1-O,2-O,6-O-Trigalloyl-beta-D-glucose;1,2,6-Trigalloylglucose | -0.140 | 0.549 | 0.615 | 0.261 | No |
| alpha-D-Galactosyl-N-acetyllactosamine | -0.134 | 0.708 | 0.742 | 0.150 | No |
| 5-Methyltetrahydropteroyltri-L-glutamate | 0.251 | 0.391 | 0.509 | 0.408 | No |
| N-Acetyl-alpha-D-glucosamine 1-phosphate | -1.167 | 0.0139 | 0.507 | 1.856 | No |
| Inositol 1,2,3,5,6-pentakisphosphate;myo-Inositol 1,2,3,5,6-pentakisphosphate;1D-myo-Inositol 1,2,3,5,6-pentakisphosphate;1L-myo-Inositol 1,2,3,4,5-pentakisphosphate | 0.252 | 0.415 | 0.521 | 0.382 | No |
| UDP-2-acetamido-4-amino-2,4,6-trideoxy-alpha-D-glucose;UDP-4-amino-4,6-dideoxy-N-acetyl-alpha-D-glucosamine;UDP-N-acetylbacillosamine | 0.311 | 0.207 | 0.509 | 0.685 | No |
| Methyl-2-alpha-L-fucopyranosyl-beta-D-galactoside | 0.905 | 0.163 | 0.509 | 0.787 | No |
| O-(1->4)-alpha-L-Dihydrostreptosyl-streptidine 6-phosphate;alpha-L-Dihydrostreptosyl-(1->4)-streptidine 6-phosphate | 1.094 | 0.0173 | 0.509 | 1.761 | No |
| Lipid X;2,3-Diacylglucosamine 1-phosphate;2,3-Bis[(3R)-3-hydroxymyristoyl]-alpha-D-glucosaminyl 1-phosphate;2,3-Bis(3-hydroxytetradecanoyl)-alpha-D-glucosaminyl 1-phosphate | -0.251 | 0.458 | 0.561 | 0.340 | No |
| alpha-D-Galactosyl-1,3-beta-D-galactosyl-1,4-N-acetyl-D-glucosamine | -0.134 | 0.708 | 0.742 | 0.150 | No |
| 1,3-alpha-D-Mannosyl-1,2-alpha-D-mannosyl-1,2-alpha-D-mannosyl-D-mannose | 0.231 | 0.387 | 0.509 | 0.412 | No |
| Taurocholate;Taurocholic acid;Cholyltaurine | -0.269 | 0.223 | 0.509 | 0.652 | No |
| GDP-3,6-dideoxy-D-galactose | -0.369 | 0.265 | 0.509 | 0.576 | No |
| **Metabolite** | **log_2_FC** | **p-value** | **FDR (BH)** | **-log_10_(p)** | **Significant (FDR < 0.05)** |
| Teleocidin B-1;(4S,7S,10S,13R)-13-Ethenyl-1,3,4,5,7,8,10,11,12,13-decahydro-4-(hydroxymethyl)-8,10,13-trimethyl-7,10-diisopropyl-6H-benzo[g][1,4]diazonino[7,6,5-cd]indol-6-one | -0.886 | 0.138 | 0.509 | 0.859 | No |
| Taurodeoxycholate;Taurodeoxycholic acid | 0.526 | 0.277 | 0.509 | 0.558 | No |
| Taurochenodeoxycholate;Taurochenodeoxycholic acid;Chenodeoxycholoyltaurine | 0.526 | 0.277 | 0.509 | 0.558 | No |
| Aerobactin | 0.215 | 0.461 | 0.561 | 0.336 | No |
| Coproporphyrin I | -0.310 | 0.296 | 0.509 | 0.529 | No |
| Coproporphyrin III | -0.310 | 0.296 | 0.509 | 0.529 | No |
| Pelargonidin;3,5,7-Trihydroxy-2-(4-hydroxyphenyl)benzopyrylium chloride;3,4',5,7-Tetrahydroxyflavylium chloride;Pelargonidin chloride;Pelargonidol chloride | 1.287 | 0.0274 | 0.509 | 1.562 | No |
| (3Z)-Phytochromobilin | 0.207 | 0.396 | 0.509 | 0.402 | No |
| Salidroside;p-Hydroxyphenethyl alcohol 1-O-beta-D-glucoside;Tyrosol glucoside | -0.400 | 0.21 | 0.509 | 0.678 | No |
| Fe-enterobactin;Fe-enterochlin | -0.214 | 0.423 | 0.525 | 0.374 | No |
| Isocarbostyril;1(2H)-Isoquinolinone;Isoquinolin-1(2H)-one | -0.652 | 0.53 | 0.605 | 0.276 | No |
| Quinolin-2-ol;2-Quinolone;2-Hydroxyquinoline;2-Quinolinol | -0.652 | 0.53 | 0.605 | 0.276 | No |
| 4-Hydroxyquinoline;Quinolin-4-ol | -0.652 | 0.53 | 0.605 | 0.276 | No |
| Benzophenone;Diphenyl ketone | -0.420 | 0.253 | 0.509 | 0.597 | No |
| Lacto-N-tetraose;beta-D-Gal-(1->3)-beta-D-GlcNAc-(1->3)-beta-D-Gal-(1->4)-D-Glc | 0.362 | 0.364 | 0.509 | 0.439 | No |
| N-Acetyl-D-galactosamine 6-phosphate | -1.167 | 0.0139 | 0.507 | 1.856 | No |
| ADP-D-glycero-beta-D-manno-heptose | -0.320 | 0.294 | 0.509 | 0.532 | No |
| ADP-L-glycero-beta-D-manno-heptose | -0.320 | 0.294 | 0.509 | 0.532 | No |
| 2(1H)-Quinolinone;Quinolin-2(1H)-one | -0.652 | 0.53 | 0.605 | 0.276 | No |
| Leukotriene F4;LTF4 | 0.207 | 0.396 | 0.509 | 0.402 | No |
| Erythronolide B | -0.460 | 0.165 | 0.509 | 0.781 | No |
| Fluoren-9-ol;9-Fluorenol;9-Hydroxyfluorene;Diphenylene carbinol | -0.420 | 0.253 | 0.509 | 0.597 | No |
| cis-1,2-Dihydroxy-1,2-dihydrodibenzothiophene | 0.394 | 0.393 | 0.509 | 0.406 | No |
| Glucosyl-limonin;Limonin 17-beta-D-glucoside | 0.275 | 0.34 | 0.509 | 0.468 | No |
| Hemine;Hemin | -0.163 | 0.656 | 0.711 | 0.183 | No |
| **Metabolite** | **log_2_FC** | **p-value** | **FDR (BH)** | **-log_10_(p)** | **Significant (FDR < 0.05)** |
| Pradimicinone I | 0.216 | 0.443 | 0.546 | 0.354 | No |
| 7-Methoxypradimicinone II | -0.445 | 0.36 | 0.509 | 0.444 | No |
| Pradimicin B | -0.349 | 0.391 | 0.509 | 0.408 | No |
| Dexylosylbenanomicin A | 0.365 | 0.279 | 0.509 | 0.555 | No |
| Amikacin | 0.592 | 0.39 | 0.509 | 0.409 | No |
| Bromocriptine | -0.451 | 0.317 | 0.509 | 0.499 | No |
| Cefonicid | 0.526 | 0.14 | 0.509 | 0.853 | No |
| Digitoxin | 0.452 | 0.182 | 0.509 | 0.740 | No |
| N-Acetyl-O-demethylpuromycin-5'-phosphate | 0.393 | 0.2 | 0.509 | 0.700 | No |
| Zafirlukast | 0.386 | 0.345 | 0.509 | 0.462 | No |
| dTDP-D-fucose;dTDP-alpha-D-fucopyranose | 0.415 | 0.23 | 0.509 | 0.638 | No |
| Pyrantel pamoate | -0.332 | 0.227 | 0.509 | 0.644 | No |
| Paramethadione | -0.486 | 0.148 | 0.509 | 0.829 | No |
| Pancuronium | -0.495 | 0.353 | 0.509 | 0.453 | No |
| Cefpodoxime proxetil | 0.348 | 0.386 | 0.509 | 0.414 | No |
| Clomiphene citrate;Clomifene citrate | -0.176 | 0.566 | 0.63 | 0.247 | No |
| Neostigmine methylsulfate | 0.793 | 0.0918 | 0.509 | 1.037 | No |
| Sodium 2-O-L-rhamnopyranosyl-4-deoxy-alpha-L-threo-hex-4-eno-pyranosiduronate;Lepidimoide | 0.653 | 0.0751 | 0.509 | 1.124 | No |
| Lychnose;1F-alpha-D-Galactosylraffinose | 0.231 | 0.387 | 0.509 | 0.412 | No |
| Sesamose;alpha-D-Galactosyl-(1-6)-alpha-D-galactosyl-(1-6)-beta-D-fructosyl-(2-1)-alpha-D-glucoside | 0.231 | 0.387 | 0.509 | 0.412 | No |
| Anthemis glycoside B | 0.223 | 0.646 | 0.704 | 0.190 | No |
| Carlina oxide | -0.420 | 0.253 | 0.509 | 0.597 | No |
| Indole-3-carboxaldehyde;Indole-3-carbaldehyde | -0.652 | 0.53 | 0.605 | 0.276 | No |
| Purpureacin-1 | -0.123 | 0.753 | 0.776 | 0.123 | No |
| Bracteatin | 1.170 | 0.0673 | 0.509 | 1.172 | No |
| Betulinic acid | -0.435 | 0.179 | 0.509 | 0.746 | No |
| Cyanidin 3-O-rutinoside;Cyanidin 3-O-rhamnosylglucoside;Keracyanin | 1.475 | 0.321 | 0.509 | 0.493 | No |
| alpha-Elemolic acid | -0.435 | 0.179 | 0.509 | 0.746 | No |
| Luteolinidin | 1.287 | 0.0274 | 0.509 | 1.562 | No |
| Pelargonin;Pelargonidin 3,5-di-beta-D-glucoside | 1.475 | 0.321 | 0.509 | 0.493 | No |
| Bruceantin | -0.611 | 0.11 | 0.509 | 0.957 | No |
| Bruceantinol | -0.187 | 0.539 | 0.613 | 0.268 | No |
| Cucurbitacin B | -0.562 | 0.42 | 0.523 | 0.377 | No |
| **Metabolite** | **log_2_FC** | **p-value** | **FDR (BH)** | **-log_10_(p)** | **Significant (FDR < 0.05)** |
| alpha-Antiarin;Antiarigenin 3-O-beta-D-antiaroside | 0.489 | 0.143 | 0.509 | 0.843 | No |
| Labriformidin | 0.270 | 0.487 | 0.574 | 0.313 | No |
| Labriformin | -1.152 | 0.0683 | 0.509 | 1.166 | No |
| Sarmentoloside;Sarmentologenin 3-O-(6-deoxy-alpha-L-taloside) | 0.207 | 0.396 | 0.509 | 0.402 | No |
| Astrasieversianin XVI | 0.656 | 0.101 | 0.509 | 0.996 | No |
| Mulberrofuran C | 0.238 | 0.679 | 0.72 | 0.168 | No |
| Gypenoside XXV | 0.656 | 0.101 | 0.509 | 0.996 | No |
| Ursolic acid | -0.435 | 0.179 | 0.509 | 0.746 | No |
| Bonafousine | 0.654 | 0.132 | 0.509 | 0.878 | No |
| Kansuinine B | -0.297 | 0.378 | 0.509 | 0.423 | No |
| Mascaroside | 0.215 | 0.461 | 0.561 | 0.336 | No |
| Ergine;Lysergamide | 1.091 | 0.209 | 0.509 | 0.680 | No |
| Osthol | -0.431 | 0.327 | 0.509 | 0.485 | No |
| Arnicolide A;Dihydrohelenalin acatate | 0.489 | 0.347 | 0.509 | 0.459 | No |
| Confertiflorin;(+)-Confertiflorin | 0.489 | 0.347 | 0.509 | 0.459 | No |
| Encelin | -0.431 | 0.327 | 0.509 | 0.485 | No |
| Eupaformonin | 0.489 | 0.347 | 0.509 | 0.459 | No |
| Gaillardin | 0.489 | 0.347 | 0.509 | 0.459 | No |
| Ipecoside | -0.162 | 0.631 | 0.696 | 0.200 | No |
| Isotenulin | 0.489 | 0.347 | 0.509 | 0.459 | No |
| Ligulatin B;Incanin | 0.489 | 0.347 | 0.509 | 0.459 | No |
| Lipiferolide | 0.489 | 0.347 | 0.509 | 0.459 | No |
| Matricin | 0.489 | 0.347 | 0.509 | 0.459 | No |
| Ovatifolin | 0.489 | 0.347 | 0.509 | 0.459 | No |
| Provincialin | 0.307 | 0.403 | 0.513 | 0.395 | No |
| Pyrethrosin | 0.489 | 0.347 | 0.509 | 0.459 | No |
| Tenulin | 0.489 | 0.347 | 0.509 | 0.459 | No |
| Vernomygdin | -0.266 | 0.349 | 0.509 | 0.457 | No |
| Viscidulin B | 0.489 | 0.347 | 0.509 | 0.459 | No |
| Xanthinin | 0.489 | 0.347 | 0.509 | 0.459 | No |
| Xanthumin | 0.489 | 0.347 | 0.509 | 0.459 | No |
| Zexbrevin B | -0.266 | 0.349 | 0.509 | 0.457 | No |
| 7-Hydroxyflavan | -0.431 | 0.327 | 0.509 | 0.485 | No |
| Manniflavanone | 0.311 | 0.207 | 0.509 | 0.685 | No |
| Allamandin | -1.165 | 0.143 | 0.509 | 0.843 | No |
| Dolichodial;Iridial | -0.420 | 0.253 | 0.509 | 0.597 | No |
| **Metabolite** | **log_2_FC** | **p-value** | **FDR (BH)** | **-log_10_(p)** | **Significant (FDR < 0.05)** |
| Naringin;Naringenin 7-O-neohesperidoside;Naringenin 7-O-[alpha-L-rhamnosyl-(1->2)-beta-D-glucoside] | 0.238 | 0.679 | 0.72 | 0.168 | No |
| Nepetalactone cis-trans-form | -0.420 | 0.253 | 0.509 | 0.597 | No |
| Nepetalactone trans-cis-form | -0.420 | 0.253 | 0.509 | 0.597 | No |
| Narirutin;Naringenin 7-O-rutinoside | 0.238 | 0.679 | 0.72 | 0.168 | No |
| Sanggenon C | -0.349 | 0.391 | 0.509 | 0.408 | No |
| Sanggenon D | -0.349 | 0.391 | 0.509 | 0.408 | No |
| Carvone oxide | -0.420 | 0.253 | 0.509 | 0.597 | No |
| beta-Citronellol;(R)-(+)-Citronellol | -0.006 | 0.991 | 0.991 | 0.004 | No |
| Piquerol A | -0.420 | 0.253 | 0.509 | 0.597 | No |
| Rotundifolone;Lippione | -0.420 | 0.253 | 0.509 | 0.597 | No |
| Karwinaphthol B | 0.489 | 0.347 | 0.509 | 0.459 | No |
| Karwinskione | 0.300 | 0.268 | 0.509 | 0.571 | No |
| Adouetine Y | 0.152 | 0.484 | 0.573 | 0.315 | No |
| Aralionine A | 0.320 | 0.541 | 0.613 | 0.267 | No |
| Crenatine A | 0.152 | 0.484 | 0.573 | 0.315 | No |
| Hymenocardine | 0.229 | 0.46 | 0.561 | 0.338 | No |
| Apigenin 7-(6''-malonylglucoside);Apigenin 7-O-(6-malonyl-beta-D-glucoside) | -0.408 | 0.284 | 0.509 | 0.547 | No |
| 6-Hydroxykaempferol | 1.170 | 0.0673 | 0.509 | 1.172 | No |
| 6-Hydroxyluteolin;5,6,7,3',4'-Pentahydroxyflavone | 1.170 | 0.0673 | 0.509 | 1.172 | No |
| Hypolaetin;8-Hydroxyluteolin | 1.170 | 0.0673 | 0.509 | 1.172 | No |
| Isoetin;5,7,2',4',5'-Pentahydroxyflavone | 1.170 | 0.0673 | 0.509 | 1.172 | No |
| Morin;3,5,7,2',4'-Pentahydroxyflavone;C.I.Natural Yellow 8 | 1.170 | 0.0673 | 0.509 | 1.172 | No |
| Robinetin;5-Deoxymyricetin | 1.170 | 0.0673 | 0.509 | 1.172 | No |
| Sciadopitysin | -0.140 | 0.549 | 0.615 | 0.261 | No |
| Tricetin;5,7,3',4',5'-Pentahydroxyflavone | 1.170 | 0.0673 | 0.509 | 1.172 | No |
| Violanthin | -1.143 | 0.157 | 0.509 | 0.805 | No |
| Terminalin;Gallagic acid | 0.897 | 0.257 | 0.509 | 0.590 | No |
| Batatasin IV;3-[2-(2-Hydroxyphenyl)ethyl]-5-methoxyphenol | -0.431 | 0.327 | 0.509 | 0.485 | No |
| 3-Hydroxy-5-methoxy-6-prenylstilbene-2-carboxylic acid | -0.408 | 0.109 | 0.509 | 0.961 | No |
| Pinosylvin methyl ether;5-Methoxy-3-stilbenol | -0.431 | 0.327 | 0.509 | 0.485 | No |
| Auriculine | -0.712 | 0.139 | 0.509 | 0.856 | No |
| **Metabolite** | **log_2_FC** | **p-value** | **FDR (BH)** | **-log_10_(p)** | **Significant (FDR < 0.05)** |
| Cascaroside A | 0.238 | 0.679 | 0.72 | 0.168 | No |
| Deoxylapachol | -0.431 | 0.327 | 0.509 | 0.485 | No |
| Cynometrine | 1.091 | 0.209 | 0.509 | 0.680 | No |
| Maytansine | -0.966 | 0.146 | 0.509 | 0.835 | No |
| (-)-Tylocrebrine | 0.389 | 0.143 | 0.509 | 0.843 | No |
| Tylophorine | 0.389 | 0.143 | 0.509 | 0.843 | No |
| Argyrolobine | -0.400 | 0.21 | 0.509 | 0.678 | No |
| Calystegin B2;Calystegine B2 | -0.486 | 0.148 | 0.509 | 0.829 | No |
| Podorhizol beta-D-glucoside | -0.332 | 0.227 | 0.509 | 0.644 | No |
| Bentazone | -0.379 | 0.236 | 0.509 | 0.626 | No |
| Amitraz;Mitac | 0.805 | 0.215 | 0.509 | 0.668 | No |
| SDS;Sodium lauryl sulfate;Sodium dodecyl sulfate | 0.489 | 0.347 | 0.509 | 0.459 | No |
| 1-Diphosinositol pentakisphosphate;D-myo-Inositol, 2,3,4,5,6-pentakis(dihydrogen phosphate) 1-(trihydrogen diphosphate);Diphospho-myo-inositol pentakisphosphate;1D-myo-Inositol 1-diphosphate 2,3,4,5,6-pentakisphosphate | -0.167 | 0.714 | 0.744 | 0.146 | No |
| Bacteriochlorophyll a | 0.522 | 0.0975 | 0.509 | 1.011 | No |
| 6alpha-Glucuronosylhyodeoxycholate | 0.453 | 0.457 | 0.561 | 0.340 | No |
| Oxolinic acid | -1.167 | 0.0139 | 0.507 | 1.856 | No |
| (-)-Citronellol | -0.006 | 0.991 | 0.991 | 0.004 | No |
| dTDP-4-dimethylamino-4,6-dideoxy-5-C-methyl-L-mannose | -0.315 | 0.351 | 0.509 | 0.454 | No |
| Methyl 2-(4-isopropyl-4-methyl-5-oxo-2-imidazolin-2-yl)-p-toluate | -0.587 | 0.179 | 0.509 | 0.748 | No |
| Methyl 6-(4-isopropyl-4-methyl-5-oxo-2-imidazolin-2-yl)-m-toluate | -0.587 | 0.179 | 0.509 | 0.748 | No |
| 5-PP-InsP5;5-Diphosphoinositol pentakisphosphate;1D-myo-Inositol 5-diphosphate pentakisphosphate;1D-myo-Inositol 5-diphosphate 1,2,3,4,6-pentakisphosphate | -0.167 | 0.714 | 0.744 | 0.146 | No |
| 15,16-Dihydrobiliverdin;15,16-Dihydrobiliverdin IXalpha;15,16-Dihydrobiliverdin ixa | 0.207 | 0.396 | 0.509 | 0.402 | No |
| 11-Hydroxyiridodial glucoside pentaacetate | 0.410 | 0.274 | 0.509 | 0.563 | No |
| 8-epi-11-Hydroxyiridodial glucoside pentaacetate | 0.410 | 0.274 | 0.509 | 0.563 | No |
| **Metabolite** | **log_2_FC** | **p-value** | **FDR (BH)** | **-log_10_(p)** | **Significant (FDR < 0.05)** |
| dTDP-D-desosamine;dTDP-3-dimethylamino-3,4,6-trideoxy-D-glucose;dTDP-alpha-D-desosamine;dTDP-3-dimethylamino-3,4,6-trideoxy-alpha-D-glucose | 0.307 | 0.21 | 0.509 | 0.677 | No |
| dTDP-6-deoxy-D-allose;dTDP-6-deoxy-alpha-D-allose | 0.415 | 0.23 | 0.509 | 0.638 | No |
| dTDP-L-megosamine | 0.307 | 0.21 | 0.509 | 0.677 | No |
| Perillic acid | -0.420 | 0.253 | 0.509 | 0.597 | No |
| Myrtenic acid;6,6-Dimethylbicyclo[3.1.1]hept-2-ene-2-carboxylic acid | -0.420 | 0.253 | 0.509 | 0.597 | No |
| Avermectin A2b monosaccharide | 0.452 | 0.182 | 0.509 | 0.740 | No |
| Avermectin A2b | 0.619 | 0.384 | 0.509 | 0.416 | No |
| 5-Oxoavermectin ''1b'' aglycone | 0.152 | 0.484 | 0.573 | 0.315 | No |
| Avermectin B2a monosaccharide | 0.452 | 0.182 | 0.509 | 0.740 | No |
| Avermectin B2a | 0.619 | 0.384 | 0.509 | 0.416 | No |
| 5-Oxoavermectin ''1a'' aglycone | 0.654 | 0.132 | 0.509 | 0.878 | No |
| 5-O-beta-D-Mycaminosyltylactone | -0.254 | 0.308 | 0.509 | 0.511 | No |
| Soyasaponin II | 0.656 | 0.101 | 0.509 | 0.996 | No |
| 3-Methoxy-8,9-methylenedioxy-3,4-dihydrophenanthridine;3-Methoxy-3,4-dihydro-[1,3]-dioxolo-[4,5-j]-phenanthridine | 1.287 | 0.0274 | 0.509 | 1.562 | No |
| Decanal;1-Decanal | -0.006 | 0.991 | 0.991 | 0.004 | No |
| 100-2 | -0.168 | 0.632 | 0.696 | 0.199 | No |
| Novclobiocin 104 | -0.336 | 0.473 | 0.569 | 0.325 | No |
| Pheniramine maleate | 1.280 | 0.0625 | 0.509 | 1.204 | No |
| Novobiocin sodium | -0.886 | 0.227 | 0.509 | 0.643 | No |
| Apigenin 7-O-neohesperidoside;Rhoifolin;Rhoifoloside | -1.143 | 0.157 | 0.509 | 0.805 | No |
| Vitexin 2''-O-beta-L-rhamnoside;2''-O-Rhamnosylvitexin;Vitexin 2''-rhamnoside | -1.143 | 0.157 | 0.509 | 0.805 | No |
| Quercetin 3-O-[beta-D-xylosyl-(1->2)-beta-D-glucoside] | 0.131 | 0.654 | 0.711 | 0.184 | No |
| Quercetin 3-O-(6-O-malonyl-beta-D-glucoside);Quercetin 3-O-malonylglucoside;Quercetin-3-O-(6''-malonylglucoside) | 0.276 | 0.306 | 0.509 | 0.514 | No |
| Carpipramine maleate | 0.231 | 0.529 | 0.605 | 0.277 | No |
| N2-Acetyl-L-aminoadipyl-delta-phosphate | -1.167 | 0.0139 | 0.507 | 1.856 | No |
| **Metabolite** | **log_2_FC** | **p-value** | **FDR (BH)** | **-log_10_(p)** | **Significant (FDR < 0.05)** |
| Ceftiofur sodium | -0.985 | 0.0756 | 0.509 | 1.121 | No |
| Kad 1229;Mitiglinide calcium | -0.419 | 0.498 | 0.586 | 0.302 | No |
| Hydrocortisone caproate;[2-(11,17-Dihydroxy-10,13-dimethyl-3-oxo-1,2,6,7,8,9,10,11,12,13,14,15,16,17-tetradecahydrocyclopenta[a]phenanthren-17-yl)-2-oxo-ethyl]hexanoate | 0.405 | 0.224 | 0.509 | 0.649 | No |
| Polidocanol;Nonaethylene glycol monododecyl ether | -0.136 | 0.743 | 0.768 | 0.129 | No |
| 5-Hydroxybisphenol A;5-Hydroxybisphenol;BPAcatechol | -0.431 | 0.327 | 0.509 | 0.485 | No |
| 1,2-Bis(4-hydroxyphenyl)-2-propanol | -0.431 | 0.327 | 0.509 | 0.485 | No |
| 2,2-Bis(4-hydroxyphenyl)-1-propanol | -0.431 | 0.327 | 0.509 | 0.485 | No |
| 4,4'-Dihydroxy-alpha-methylstilbene | -0.431 | 0.327 | 0.509 | 0.485 | No |
| Batrachotoxin | 0.348 | 0.284 | 0.509 | 0.547 | No |
| 1-Hexadecanoyl-2-(9Z-octadecenoyl)-sn-glycero-3-phosphonoethanolamine | -0.410 | 0.381 | 0.509 | 0.420 | No |
| Diphenhydramine salicylate | 0.389 | 0.143 | 0.509 | 0.843 | No |
| UR-12947 | -0.154 | 0.577 | 0.641 | 0.239 | No |
| 5-Carboxy-2-pentenoyl-CoA;2,3-Dehydroadipyl-CoA | 0.788 | 0.0644 | 0.509 | 1.191 | No |
| Cyromazine;Cyclopropylmelamine;2-Cyclopropylamino-4,6-diamino-s-triazine | -0.420 | 0.253 | 0.509 | 0.597 | No |
| Acetrizoic acid | 0.459 | 0.111 | 0.509 | 0.955 | No |
| 3,3'-Dimethylbisphenol A;2,2-Bis(4-hydroxy-3-methylphenyl)propane | 0.227 | 0.142 | 0.509 | 0.848 | No |
| 4-Butoxyphenol | -0.420 | 0.253 | 0.509 | 0.597 | No |
| 2-Hydroxyfluorene;Fluoren-2-ol | -0.420 | 0.253 | 0.509 | 0.597 | No |
| Bisphenol A glycidylmethacrylate;Bis-GMA;2,2-Bis[4-(2-hydroxy-3-methacryloxypropoxy)phenyl]propane;Bisphenol A diglycidyl methacrylate | 0.300 | 0.268 | 0.509 | 0.571 | No |
| Camphorquinone | -0.420 | 0.253 | 0.509 | 0.597 | No |
| Chlorobenzilate;Ethyl-4,4'-dichlorobenzilate;Ethyl 2-hydroxy-2,2-bis(4-chlorophenyl)acetate | -0.579 | 0.0725 | 0.509 | 1.139 | No |
| **Metabolite** | **log_2_FC** | **p-value** | **FDR (BH)** | **-log_10_(p)** | **Significant (FDR < 0.05)** |
| (E)-1-Methoxy-4-[2-(4-nitrophenyl)ethenyl]benzene;(E)-Methoxy-4'-nitrostilbene | 1.287 | 0.0274 | 0.509 | 1.562 | No |
| BMS 379224 | 0.560 | 0.0585 | 0.509 | 1.233 | No |
| 9-Hydroxybenzo[a]pyrene-4,5-oxide;9-Hydroxybenzo[a]pyrene-4,5-epoxide | 0.235 | 0.308 | 0.509 | 0.512 | No |
| 4,5-Dihydro-4-hydroxy-5-S-glutathionyl-benzo[a]pyrene | -0.073 | 0.802 | 0.818 | 0.096 | No |
| 7,8-Dihydro-7-hydroxy-8-S-glutathionyl-benzo[a]pyrene | -0.073 | 0.802 | 0.818 | 0.096 | No |
| 2-(4-Hydroxyphenyl)-5,6,7,8-tetrahydroxy-4H-1-benzopyran-4-one;5,6,7,8,4'-Pentahydroxyflavone;Nortangeretin | 1.170 | 0.0673 | 0.509 | 1.172 | No |
| 2,2,4-Trimethyl-3-(4-methoxyphenyl)-2H-1-benzopyran-7-ol acetate | -0.408 | 0.109 | 0.509 | 0.961 | No |
| alpha-(p-Methoxyphenyl)-4-pyridineacrylic acid | 1.287 | 0.0274 | 0.509 | 1.562 | No |
| 3,3-Bis(4-hydroxyphenyl)pentane;4,4'-(1-Ethylpropane-1,1,-diyl)diphenol | 0.227 | 0.142 | 0.509 | 0.848 | No |
| Taurohyocholate;N-(3alpha,6alpha,7alpha-Trihydroxy-5beta-cholan-24-oyl)taurine | -0.269 | 0.223 | 0.509 | 0.652 | No |
| GW 6471 | 0.414 | 0.476 | 0.572 | 0.322 | No |
| GW 1929 | 0.482 | 0.241 | 0.509 | 0.618 | No |
| SR 12813;GW 485801 | 0.660 | 0.159 | 0.509 | 0.799 | No |
| GW 3965 | 0.272 | 0.299 | 0.509 | 0.524 | No |
| Onnamide A;Onnamide | 0.343 | 0.581 | 0.644 | 0.236 | No |
| Spirilloxanthin;Rhodoviolascin | 0.488 | 0.214 | 0.509 | 0.669 | No |
| Hydroxyspheroidenone | 0.632 | 0.304 | 0.509 | 0.517 | No |
| GDP-L-gulose | 0.590 | 0.345 | 0.509 | 0.462 | No |
| L-Galactonate;L-Galactonic acid | 0.394 | 0.393 | 0.509 | 0.406 | No |
| (3R,2'S)-Myxol 2'-(2,4-di-O-methyl-alpha-L-fucoside) | -0.604 | 0.13 | 0.509 | 0.887 | No |
| Thermocryptoxanthin-13 | 0.809 | 0.0821 | 0.509 | 1.086 | No |
| myo-Inositol pentakisphosphate | 0.252 | 0.415 | 0.521 | 0.382 | No |
| Malonylgenistin | -0.408 | 0.284 | 0.509 | 0.547 | No |
| Pelargonidin 3-O-3'',6''-O-dimalonylglucoside | -0.315 | 0.351 | 0.509 | 0.454 | No |
| Pelargonidin 3-O-sophoroside | 1.475 | 0.321 | 0.509 | 0.493 | No |
| Pelargonidin 3-O-beta-D-glucoside 5-O-(6-coumaroyl-beta-D-glucoside) | -0.153 | 0.705 | 0.742 | 0.152 | No |
| **Metabolite** | **log_2_FC** | **p-value** | **FDR (BH)** | **-log_10_(p)** | **Significant (FDR < 0.05)** |
| Delphinidin 3-O-beta-D-glucoside 5-O-(6-coumaroyl-beta-D-glucoside) | 0.223 | 0.646 | 0.704 | 0.190 | No |
| 1,7-Dimethyluric acid | 0.394 | 0.393 | 0.509 | 0.406 | No |
| 3,7-Dimethyluric acid | 0.394 | 0.393 | 0.509 | 0.406 | No |
| Delphinidin 3-O-(6-caffeoyl-beta-D-glucoside) | 0.500 | 0.356 | 0.509 | 0.449 | No |
| Cyanidin 3-glucoside 5-caffeoylglucoside | 0.223 | 0.646 | 0.704 | 0.190 | No |
| 4-Amino-2-hydroxylamino-6-nitrotoluene | 0.531 | 0.237 | 0.509 | 0.626 | No |
| Naringin chalcone | 0.238 | 0.679 | 0.72 | 0.168 | No |
| 7(1)-Hydroxychlorophyllide a | -0.446 | 0.234 | 0.509 | 0.630 | No |
| NPC | 0.307 | 0.403 | 0.513 | 0.395 | No |
| Homotrypanothione disulfide | 1.054 | 0.063 | 0.509 | 1.201 | No |
| Deoxypumiloside | -1.340 | 0.114 | 0.509 | 0.942 | No |
| AM-toxin I | -0.444 | 0.195 | 0.509 | 0.710 | No |
| Cascaroside B | 0.238 | 0.679 | 0.72 | 0.168 | No |
| Glucofrangulin A | -1.143 | 0.157 | 0.509 | 0.805 | No |
| Tauroursodeoxycholic acid | 0.526 | 0.277 | 0.509 | 0.558 | No |
| S 1319 | -0.379 | 0.236 | 0.509 | 0.626 | No |
| Atractylodin | -0.420 | 0.253 | 0.509 | 0.597 | No |
| Atractylone;Atractylol;Atractyloxide | 0.227 | 0.142 | 0.509 | 0.848 | No |
| Furanodiene | 0.227 | 0.142 | 0.509 | 0.848 | No |
| Kaempferitrin;Kaempferol-dirhamnoside;Lespenefril | -1.143 | 0.157 | 0.509 | 0.805 | No |
| Callytriol C | -0.266 | 0.349 | 0.509 | 0.457 | No |
| Ophiopogonin A | 0.452 | 0.182 | 0.509 | 0.740 | No |
| Oleanolic acid;3beta-Hydroxyolean-12-en-28-oic acid;Astrantiagenin C;Caryophyllin | -0.435 | 0.179 | 0.509 | 0.746 | No |
| 2-Oxo-8-methylthiooctanoic acid;8-Methylthio-2-octanoic acid | 0.523 | 0.0167 | 0.509 | 1.779 | No |
| 2-Hydroxyethylclavam;Hydroxyethylclavam | -0.486 | 0.148 | 0.509 | 0.829 | No |
| Soyasapogenol E | -0.435 | 0.179 | 0.509 | 0.746 | No |
| Linderalactone | -0.431 | 0.327 | 0.509 | 0.485 | No |
| Kanokoside D | 0.152 | 0.484 | 0.573 | 0.315 | No |
| di-trans,poly-cis-Pentaprenyl diphosphate;ditrans,dicis-Pentaprenyl diphosphate;2-cis,6-cis,10-trans,14-trans-Pentaprenyl diphosphate;2-cis,6-cis,10-trans,14-trans-Geranylfarnesyl diphosphate | 0.410 | 0.274 | 0.509 | 0.563 | No |
| Gallotannin;1,3,6-Tri-o-galloyl-beta-D-glucose | -0.140 | 0.549 | 0.615 | 0.261 | No |
| **Metabolite** | **log_2_FC** | **p-value** | **FDR (BH)** | **-log_10_(p)** | **Significant (FDR < 0.05)** |
| Metoxadiazone | -0.420 | 0.253 | 0.509 | 0.597 | No |
| Akeboside Stf | 0.656 | 0.101 | 0.509 | 0.996 | No |
| Zizybeoside II | -0.886 | 0.227 | 0.509 | 0.643 | No |
| Ubiquinone-6;Coenzyme Q6 | -0.067 | 0.847 | 0.858 | 0.072 | No |
| (6E)-8-Oxogeranial;10-Oxogeranial;8-Oxogeranial | -0.420 | 0.253 | 0.509 | 0.597 | No |
| Schizonepetoside E | -0.266 | 0.349 | 0.509 | 0.457 | No |
| 4-(4-Hydroxyphenyl)-2-butanol;4-HPB;Rhododendrol;Betuligenol | -0.420 | 0.253 | 0.509 | 0.597 | No |
| Gentamicin X2 | 0.405 | 0.224 | 0.509 | 0.649 | No |
| Ethadione | -0.486 | 0.148 | 0.509 | 0.829 | No |
| 3-Methoxytyramine-betaxanthin | -0.408 | 0.109 | 0.509 | 0.961 | No |
| (+/-)-Asarinol A | -0.420 | 0.253 | 0.509 | 0.597 | No |
| Eriojaposide A | 0.307 | 0.403 | 0.513 | 0.395 | No |
| Cannabisin B | 0.588 | 0.0664 | 0.509 | 1.178 | No |
| Cannabisin D | 0.207 | 0.396 | 0.509 | 0.402 | No |
| Cannabisin F | 0.207 | 0.396 | 0.509 | 0.402 | No |
| Cannabisin G | 0.207 | 0.396 | 0.509 | 0.402 | No |
| Thiazolylethylamine;2-(2-Aminoethyl)thiazole | -0.394 | 0.0196 | 0.509 | 1.709 | No |
| Clobenpropit | -1.165 | 0.143 | 0.509 | 0.843 | No |
| Formimidoyl-fortimicin A;Dactimicin;SF-2052 | 0.297 | 0.0677 | 0.509 | 1.169 | No |
| 1-Epidactimicin | 0.297 | 0.0677 | 0.509 | 1.169 | No |
| Pheophorbide a | -0.446 | 0.234 | 0.509 | 0.630 | No |
| dTDP-D-angolosamine | 0.307 | 0.21 | 0.509 | 0.677 | No |
| N-Acetyl-alpha-D-hexosamine 1-phosphate | -1.167 | 0.0139 | 0.507 | 1.856 | No |
| N-Acetyl-alpha-D-galactosamine 1-phosphate | -1.167 | 0.0139 | 0.507 | 1.856 | No |
| C-13(2)-Carboxypyropheophorbide a;C-13(2)-Carboxylpyropheophorbide a | -0.886 | 0.227 | 0.509 | 0.643 | No |
| N1,N5-Tri-di(hydroxyferuloyl)-N10-sinapoyl-spermidine | 1.054 | 0.063 | 0.509 | 1.201 | No |
| Tributyltin oxide;TBTO | 0.650 | 0.147 | 0.509 | 0.833 | No |
| Bacteriochlorophyllide b | -0.446 | 0.234 | 0.509 | 0.630 | No |
| 2,2',4,5'-Tetrabromodiphenyl ether;PBDE 49;BDE 49 | -0.199 | 0.724 | 0.75 | 0.140 | No |
| 2,2',4,4'-Tetrabromodiphenyl ether;PBDE 47;BDE 47 | -0.199 | 0.724 | 0.75 | 0.140 | No |
| 9,10-Dihydroxybenzo[a]pyrene;Benzo[a]pyrene-9,10-diol | 0.235 | 0.308 | 0.509 | 0.512 | No |
| **Metabolite** | **log_2_FC** | **p-value** | **FDR (BH)** | **-log_10_(p)** | **Significant (FDR < 0.05)** |
| 4,5-Dihydroxybenzo[a]pyrene;Benzo[a]pyrene-4,5-diol | 0.235 | 0.308 | 0.509 | 0.512 | No |
| 11,12-Dihydroxybenzo[a]pyrene;Benzo[a]pyrene-11,12-diol | 0.235 | 0.308 | 0.509 | 0.512 | No |
| 4-n-Butylresorcinol;Rucinol (TN) | -0.420 | 0.253 | 0.509 | 0.597 | No |
| Oxocamphor;10-Oxocamphor;beta-Oxocamphor | -0.420 | 0.253 | 0.509 | 0.597 | No |
| Fosthiazate | -1.167 | 0.0139 | 0.507 | 1.856 | No |
| Tralomethrin | -1.038 | 0.165 | 0.509 | 0.782 | No |
| Oxadiazon | 0.653 | 0.0751 | 0.509 | 1.124 | No |
| Indoxacarb | 0.440 | 0.276 | 0.509 | 0.559 | No |
| Bifenazate | 0.905 | 0.163 | 0.509 | 0.787 | No |
| dTDP-L-rhodosamine | 0.307 | 0.21 | 0.509 | 0.677 | No |
| Kelevan | 0.740 | 0.154 | 0.509 | 0.811 | No |
| Trichloronat | -0.386 | 0.313 | 0.509 | 0.504 | No |
| Norbormide | 0.482 | 0.241 | 0.509 | 0.618 | No |
| 1,2-Dimethylhydrazine | -0.625 | 0.148 | 0.509 | 0.831 | No |
| Tris(2,3-dibromopropyl) phosphate | -0.739 | 0.306 | 0.509 | 0.514 | No |
| 1,1-Dimethylhydrazine | -0.625 | 0.148 | 0.509 | 0.831 | No |
| Fumonisin B2 | -0.061 | 0.846 | 0.858 | 0.073 | No |
| Aziridyl benzoquinone;Benzoquinone aziridine | -0.408 | 0.109 | 0.509 | 0.961 | No |
| Cyclochlorotine | 0.500 | 0.356 | 0.509 | 0.449 | No |
| Ethyl selenac | -0.223 | 0.507 | 0.595 | 0.295 | No |
| 8-Hydroxyquinoline;Oxyquinoline | -0.652 | 0.53 | 0.605 | 0.276 | No |
| N-Methyl-N,4-dinitrosoaniline;N,4-Dinitrosomethylaniline | 0.531 | 0.237 | 0.509 | 0.626 | No |
| 6-[2,3-Dihydroxy-1-(hydroxymethyl)propyl]-1,2-dihydro-7-hydroxy-9-methoxy-cyclopenta[c][1]benzopyran-3,4-dione | 0.793 | 0.0918 | 0.509 | 1.037 | No |
| 3,4-Didehydroadipyl-CoA;cis-3,4-Dehydroadipyl-CoA | 0.788 | 0.0644 | 0.509 | 1.191 | No |
| Debromohymenialdisine;(Z)-Debromohymenialdisine;DBH | -1.167 | 0.0139 | 0.507 | 1.856 | No |
| tert-Butylbicyclophosphorothionate;tert-Butylbicyclophosphorothioic acid;TBPS | -0.379 | 0.236 | 0.509 | 0.626 | No |
| **Metabolite** | **log_2_FC** | **p-value** | **FDR (BH)** | **-log_10_(p)** | **Significant (FDR < 0.05)** |
| UDP-4-amino-4,6-dideoxy-N-acetyl-beta-L-altrosamine | 0.311 | 0.207 | 0.509 | 0.685 | No |
| Cerberin;2'-Acetylneriifolin | -0.562 | 0.42 | 0.523 | 0.377 | No |
| Oleandrin | -0.562 | 0.42 | 0.523 | 0.377 | No |
| Satratoxin H | -0.106 | 0.767 | 0.788 | 0.115 | No |
| Verruculogen;TR 1;TR 1 toxin | 0.203 | 0.564 | 0.629 | 0.249 | No |
| CMP-pseudaminic acid | -0.170 | 0.665 | 0.716 | 0.177 | No |
| N-Demethylansamitocin P-3;N-Demethyl-AP-3;PND-3 | 0.320 | 0.541 | 0.613 | 0.267 | No |
| DPDPE;2,5-Pen-enkephalin | -0.109 | 0.69 | 0.73 | 0.161 | No |
| DSLET;2-Ser-thr-leu-enkephalin;Enkephalin, Ser(2), Leu(5), Thr(6)-;[D-Ser2,Leu5]Enkephalin-Thr6 | -0.070 | 0.812 | 0.827 | 0.090 | No |
| UDP-N-acetyl-alpha-D-glucosamine 3'-phosphate | 0.432 | 0.234 | 0.509 | 0.631 | No |
| Bornane-2,6-dione;6-Oxocamphor | -0.420 | 0.253 | 0.509 | 0.597 | No |
| Lissamine rhodamine B | -0.140 | 0.549 | 0.615 | 0.261 | No |
| CMP-N,N'-diacetyllegionaminate | -0.170 | 0.665 | 0.716 | 0.177 | No |
| Pelargonidin 3,7-di-O-beta-D-glucoside | 1.475 | 0.321 | 0.509 | 0.493 | No |
| 30-Hydroxy-11-oxo-beta-amyrin | -0.435 | 0.179 | 0.509 | 0.746 | No |
| Pulcherriminic acid | 0.227 | 0.142 | 0.509 | 0.848 | No |
| Lolicine B | -0.254 | 0.428 | 0.53 | 0.368 | No |
| 20,21-Diprenylterpendole J | -0.339 | 0.372 | 0.509 | 0.429 | No |
| GDP-beta-L-colitose | -0.369 | 0.265 | 0.509 | 0.576 | No |
| O-Acetyl-ADP-ribose;2'-O-Acetyl-ADP-ribose;AADPR | -0.320 | 0.294 | 0.509 | 0.532 | No |
| dTDP-alpha-D-fucofuranose | 0.415 | 0.23 | 0.509 | 0.638 | No |
| 6''-O-Carbamoylkanamycin A | 0.934 | 0.137 | 0.509 | 0.863 | No |
| dTDP-beta-L-evernosamine | 0.307 | 0.21 | 0.509 | 0.677 | No |

p-values were calculated using independent samples t-tests between the control group (CG) and the aerobic exercise group (AEG). False discovery rate (FDR) adjustment was performed using the Benjamini–Hochberg (BH) method to account for multiple testing. Log₂ fold change (log₂FC) represents the relative abundance ratio between groups (AEG/CG). P-values are reported to three significant figures (p < 0.0001 reported as "< 0.0001"). Multiple metabolite names separated by semicolons represent alternative names or database synonyms referring to the same metabolite.
